# The genetic association of the transcription factor NPAT with glycemic response to metformin involves regulation of fuel selection

**DOI:** 10.1371/journal.pone.0253533

**Published:** 2021-07-01

**Authors:** Changwei Chen, Jennifer R. Gallagher, Jamie Tarlton, Lidy van Aalten, Susan E. Bray, Michael L. J. Ashford, Rory J. McCrimmon, Ewan R. Pearson, Alison D. McNeilly, Calum Sutherland

**Affiliations:** 1 Division of Cellular Medicine, School of Medicine, University of Dundee, Ninewells Hospital and Medical School, James Arnott Drive, Dundee, United Kingdom; 2 Division of Systems Medicine, School of Medicine, University of Dundee, Ninewells Hospital and Medical School, James Arnott Drive, Dundee, United Kingdom; 3 Tayside Tissue Bank, School of Medicine, University of Dundee, Ninewells Hospital and Medical School, James Arnott Drive, Dundee, United Kingdom; 4 Division of Population Health and Genomics, School of Medicine, University of Dundee, Ninewells Hospital and Medical School, James Arnott Drive, Dundee, United Kingdom; University of Hawai’i at Manoa College of Tropical Agriculture and Human Resources, UNITED STATES

## Abstract

The biguanide, metformin, is the first-choice therapeutic agent for type-2 diabetes, although the mechanisms that underpin metformin clinical efficacy remain the subject of much debate, partly due to the considerable variation in patient response to metformin. Identification of poor responders by genotype could avoid unnecessary treatment and provide clues to the underlying mechanism of action. GWAS identified SNPs associated with metformin treatment success at a locus containing the *NPAT* (nuclear protein, ataxia-telangiectasia locus) and *ATM* (ataxia-telangiectasia mutated) genes. This implies that gene sequence dictates a subsequent biological function to influence metformin action. Hence, we modified expression of *NPAT* in immortalized cell lines, primary mouse hepatocytes and mouse tissues, and analysed the outcomes on metformin action using confocal microscopy, immunoblotting and immunocytochemistry. In addition, we characterised the metabolic phenotype of *npat* heterozygous knockout mice and established the metformin response following development of insulin resistance. NPAT protein was localised in the nucleus at discrete loci in several cell types, but over-expression or depletion of NPAT in immortalised cell models did not change cellular responses to biguanides. In contrast, metformin regulation of respiratory exchange ratio (RER) was completely lost in animals lacking one allele of *npat*. There was also a reduction in metformin correction of impaired glucose tolerance, however no other metabolic abnormalities, or response to metformin, were found in the *npat* heterozygous mice. In summary, we provide methodological advancements for the detection of NPAT, demonstrate that minor reductions in NPAT mRNA levels (20–40%) influence metformin regulation of RER, and propose that the association between *NPAT* SNPs and metformin response observed in GWAS, could be due to loss of metformin modification of cellular fuel usage.

## Introduction

Metformin is now the only biguanide class of drug still used in clinical practice. Interestingly despite being the main drug prescribed for type 2 diabetes the key molecular mechanisms that underpin metformin efficacy are still debated. There is evidence that metformin will reduce hepatic glucose production, improve insulin sensitivity by increasing peripheral glucose uptake and utilization, and modify the gut-brain axis [1–3]. At a molecular level, metformin activates AMP-activated protein kinase (AMPK) and inhibits protein S6 kinase 1(S6K1) by targeting a variety of proteins or protein complexes such as complex 1 of the respiratory chain, REDD1 (regulated in DNA Damage and Development), RAG (RAS-Related GTP-binding protein) and MID1-alpha4-protein of a PP2A complex [1, 4–8]. This wide range of potential mechanisms may explain the considerable variation in patient response to metformin. A better understanding of the mechanisms underlying the inter-individual difference in response could help identify poor responders so that these patients would not undergo months of ineffective treatment. Similarly, this knowledge may help develop much needed alternative therapies for poor responders.

Pharmacogenomic approaches have identified genes and variants associated with the action of metformin [9, 10]. We performed a human GWAS (genome wide association study) searching for variants that influence metformin control of blood glucose in people with type 2 diabetes [11]. We identified 14 SNPs within a large block of linkage disequilibrium that included 7 genes associated with metformin treatment success. Subsequent, eQTL analysis narrowed down the genetic signal to the *NPAT* (nuclear protein, ataxia-telangiectasia locus; Uniprot code- Q14207 (NPAT_HUMAN)) and *ATM* (ataxia-telangiectasia mutated) genes, with the most convincing signal in *NPAT*. Both human *NPAT* and *ATM* genes are located on chromosomal 11. They share a 0.5 kb gene promoter sequence upstream of their respective transcriptional start sites and are transcribed in opposite directions. Hence, these genes may be co-ordinately regulated through common gene promoter elements, while it has been proposed that each gene product may directly regulate the gene transcription of the other [12–14]. This may explain why SNPs in both genes associate with metformin response. The human *NPAT* gene covers 65 kb of DNA and the major transcript of the gene consists of 18 exons encoding a protein of 1427 aa. [12, 13]. NPAT protein is involved in the acceleration of G1 phase of the cell cycle and the activation of both histone and non-histone gene transcription [15, 16]. The *ATM* gene codes for a 350 kDa protein with a structure related to PI 3-kinases [17]. It is a key component of the DNA damage response that maintains the integrity of the genome, impacts the response to oxidative stress, hypoxia and autophagy and modifies metabolic status [18]. Activating mutations in *ATM* result in ataxia telangiectasia (AT) syndrome, a disorder characterised by cerebellar ataxia and oculocutaneous telangiectasia [19]. In addition to these clinical phenotypes, AT is associated with an increased risk of diabetes and marked insulin resistance [20, 21]. Based on our GWAS data and the physical link between *ATM* and *NPAT* genes, we investigated whether *NPAT* may also modify glucose homeostasis and the response to metformin. The aim was to learn more about NPAT biology and to begin to elucidate the potential biological mechanism underlying the association of SNPs in the human *NPAT* gene with metformin control of glucose in diabetes patients.

## Material and methods

### Chemicals and reagents

Methanol and ethanol were from VWR International Ltd. (Poole, UK). Metformin (D150959), phenformin (p7045), and other general chemicals were purchased from Sigma-Aldrich (Poole, UK). ECL Western blotting analysis system (RNP2109) was from GE Healthcare (Little Chalfont Bucks). Dulbecco’s Modified Eagle Medium (DMEM) (41965–039, 31885–023), Dulbecco’s phosphate-buffered saline (DPBS) (14190), trypsin (25300–054), penicillin-streptomycin (15140122), lipofectamine 2000, Superscript VILO Master Mix (11755–050), Taqman Universal PCR Master Mix (4324020) were from Thermo-Fisher Scientific (Perth, Scotland). HiPerFect transfection reagent (301704) was from Qiagen (Manchester, UK). Microscopic glass slides (631–0906) were from VWR International (Lutterworth, UK). Vectashield hard-set anti-fade mounting medium with DAPI (H-1500) was from Vector Laboratories Inc. (Peterborough, UK). Details of antibodies (S1 Table), plasmids (S2 Table, oligos (S3 Table) and viruses (S2 Table) are listed in S1–S3 Tables.

### Construction of pcDNA5/RT/TO-NPAT plasmid (for conditional stable cell line production)

NPAT cDNA was amplified by PCR using a plasmid from Origene (RC220768) as a template. The PCR products were then extracted and purified before being ligated into the pGEM-T Easy vector according to manufacturer instructions. The full NPAT cDNA was excised from the pGEM-T Easy vector and cloned into the pcDNA5/RT/TO vector using the NotI restriction site. Plasmid sequencing was performed to confirm successful orientation and validity of full NPAT sequence.

### Generation of stable cell lines

#### Stable HEK293 cells over-expressing NPAT

The HEK293 Tet-On cell line (Flp-In T-Rex 293) purchased from Invitrogen (R780-07) was transfected with 2 μg of pcDNA5/RT/TO-NPAT and 10 μg of pOG44 using lipofectamine 2000 (Invitrogen) according to manufacturer instructions. Selection of stable cell lines was initiated 2 days after transfection using 75 μg/ml hygromycin. Twelve days after transfection, single colonies were isolated and cultured in 24-well plates. Cells were then expanded in 12-well and 6-well plates and finally in 25 cm^2^ flasks. Induced expression of NPAT in the stable cells was verified by Western blotting using antibody sc32359.

#### Stable SHSY5Y cells under-expressing NPAT

SHSY-5Y cells were a kind gift from Professor Mike Ashford, University of Dundee. MISSION shRNA lentiviral transduction particles were used to Knock down NPAT in SHSY5Y cells. TRC1.5-pLKO.1-puro vector containing a hairpin insert with gene-specific sequence was used for cell transduction according to manufacturer protocols, in addition to hexadimethrine bromide (8μg/ml), to enhance transduction efficiency. Sequences of inserts in shRNA constructs targeting the NPAT gene are in S2 Table. MISSION PLKO.1-Puro-CMV-TurboGFP transduction particles (SHC003V) were used to monitor transduction efficiency. Transduced SHSY5Y cells were selected with 1 μg/ml puromycin and single cells were then plated in individual wells of a 96-well plate. Cells were expanded and the knockdown of NPAT expression was verified by Western blotting analysis.

### Culture of cell lines

All cells were grown in an incubator at 37°C and 5% CO_2_. HEK293, SHSY5Y and HepG2 cells (a gift from Professor Dario Alessi, University of Dundee) were cultured in DMEM containing 4.5g/l glucose and no sodium pyruvate supplemented with 10% FCS and 1% penicillin/streptomycin. H4IIe cells were grown in DMEM containing 5% FCS and 1% penicillin/streptomycin. Primary mouse hepatocytes were isolated and maintained as described previously [22]. Stable 293 transfectants were grown in DMEM containing 4.5 g/l glucose supplemented with 10% FCS and 1% penicillin/streptomycin, 75μg/ml hygromycin and 15 μg/ml blasticidin. Expression of NPAT was induced by culturing cells in fresh medium containing tetracycline at concentrations and times as indicated in figure legends. Stable SHSY5Y transfectants were grown in DMEM containing 4.5 g/l glucose supplemented with 10% FCS and 1% penicillin/streptomycin.

### Plasmid DNA transfection

HEK293 cells were plated in 24-well (10^5^ cells/well) or 6-well (6x10^5^ cells/well) plates and incubated at 37°C and 5% CO_2_ overnight. 4 μg (6-well) or 1 μg (24-well) of DNA was transfected per well using lipofectamine 2000 according to the manufacturer instructions. For shRNA transfection, HEK293 cells were plated in 6-well plates at a density of 2 x 10^5^ cells/well and incubated at 37°C and 5% CO_2_ overnight. siRNA were transfected at final concentration of 5 nM per well using HiperFect (Qiagen) according to manufacturer protocols. Cells were incubated for 24 hr prior to treatment or lysis for the preparation of protein/RNA.

### Preparation of protein extract from cells and tissues

Cells were washed in ice-cold PBS and depending on the procedure to follow, collected in Tris-Triton lysis buffer: 50 mM Tris-HCl, pH 7.4, 50 mM NaF, 1 mM Na pyrophosphate, 1 mM EDTA, 1 mM EGTA, 50 mM NaCl, 1% Triton X-100, 0.92% sucrose and protease inhibitors cocktail (1183617000, Roche); IP lysis buffer: 50 mM Tris-HCl, pH7.4, 150 mM NaCl, 1 mM EDTA, 1% Triton X-100 and protease inhibitors cocktail (1183617000, Roche); or RIPA buffer: 50 mM Tris-HCl, pH8, 150 mM NaCl, 1% NP40, 0.5% sodium deoxycholate, 0.1% SDS, 2 mM PMSF, 1 mM sodium orthovanadate and protease inhibitors cocktail (1183617000, Roche). Cell lysis was performed on ice for 20 min before being centrifuged for 20 min at 13000 rpm at 4°C. Supernatants were collected and the pellet was resuspended in Urea lysis buffer containing 15 mM Tris-HCl (pH 7.5), 48% urea, 8.7% glycerol, 1% SDS, 143 mM mercaptoethanol, 0.004% bromphenol blue, and protease inhibitors cocktail (P8340, Sigma). For the preparation of whole-cell extracts, cells were washed with PBS twice and then collected in Urea lysis buffer. Cells were lysed for 20 min at 4°C under constant agitation and then homogenized using QIAshredder spin columns according to manufacturer instructions. For the preparation of tissue extracts, various tissues were manually ground in liquid nitrogen using a mortar and pestle and the resulting tissue powder was suspended in Urea lysis buffer. Tissue lysis was performed for 20 min at 4°C under constant agitation and the lysates were homogenized using QIAshredder spin columns according to manufacturer instructions.

### Western blotting analysis

Cell or tissue lysates were heated at 95°C for 5 min and then centrifuged at 13,000 rpm for 5 min. Equal quantities of proteins (25μg) were separated by SDS-PAGE (6% or 4–12%), transferred to PVDF membrane, incubated in blocking solution (PBS containing 0.1% Tween-20 (PBST) and 5% non-fat milk) for 1 hr at room temperature, followed by incubation overnight at 4°C in the primary antibodies in PBST containing 1% BSA. After washing in PBST, the membranes were incubated in secondary antibodies in PBST for 1 hr at room temperature, washed with PBST, and the protein signals detected using an ECL Western blotting analysis system and Fuji Medical X-ray films. Films were scanned on a CanoScan LiDE 100 scanner and images saved as JPG files. Densitometry of protein bands was carried out using the AIDA densitometry software (Raytest).

### Quantification of mRNA expression

Total RNA from cells or mouse tissues was isolated using TRI Reagent according to manufacturer instructions. cDNA was synthesized using SuperScript VILO MasterMix kit (Invitrogen). Real time PCR was carried out with the 7900HT Fast Real-Time PCR System (Applied Biosystems) using TaqMan 2× Universal PCR Master Mix (Applied Biosystems) and primer/ probes sets as in S3 Table. Cycling conditions were as follows: 50°C for 2 min, 95°C for 10 min, followed by 40 cycles of 95°C for 15 sec and 60°C for 1 min. Relative levels of gene expression were determined using the 2^-ΔΔCT^ method with β-actin as a reference gene. Samples were analysed in triplicates.

### Microscopy

#### Immunofluorescence

Cells were cultured on coverslips pre-treated with 50 μg/ml poly-D-lysine (Sigma P6407). Cells were rinsed with PBS, fixed with 4% PFA for 10 min at room temperature, washed again with PBS and then incubated in PBS-0.5% Triton X100 for 10 min. A blocking step with 1% BSA in PBS-0.1% Tween-20 (PBST) for 30 min was carried out to minimise non-specific antibody binding, prior to incubation overnight with an anti-NPAT antibody (Cat. No. 611344, BD Bioscience) or an anti-ATM antibody (Cat. No. ab199726, Abcam) (1:250 in PBST) at 4°C. Cells were washed 3 times in PBST and incubated with a donkey anti-mouse IgG, or donkey anti-rabbit IgG, secondary antibodies (Alexa Fluor488 (GREEN), Abcam) (1:1000 in PBST). Finally, cells were rinsed 3 times with PBST and mounted on microscope slides with Vectorshield mounting medium with DAPI DNA stain (H1500, Vector Laboratories). Images were acquired using a confocal microscope (Leica SP5) with a ×40 oil immersion objective.

#### Immunohistochemistry

Antigen retrieval and de-paraffinisation was performed using DAKO EnVision^™^ FLEX Target Retrieval solution (high pH) buffer (50x conc) (K8004) in a DAKO PT Link for 20 min at 97°C. Immunostaining using DAKO EnVision^™^ FLEX system on a DAKO Autostainer Link48 was carried out according to manufacturer protocols. Sections were initially washed in Flex Wash Buffer (K8006), then Flex Peroxidase-Blocking Reagent (SM801) applied for 5 min followed by Goat serum 10%(v/v) stock avidin solution (Vector Labs) (SP-2001) for 15 min, prior to incubation with anti-NPAT primary antibody (Bethyl Labs A302-772A-M) at a dilution of 1:1000 in Flex Antibody Diluent (K8006) including 10%(v/v) from stock biotin solution (Vector Labs) (SP-2001). After washing with Flex Wash Buffer the Biotinylated anti-rabbit antibody was added for 30 min and Vectastain^®^ Elite ABC reagent for a further 30 min. Next the slides were incubated with Flex DAB+ working solution (SM803) for 2 x 5 min, Copper Sulphate solution for 5 min, then Flex Haematoxylin (SM806) for 5 min. In between each step, sections were rinsed with Flex Wash Buffer with a final wash in dH_2_O. The same protocol was used for the primary anti-ATM (Abcam EPR17059 ab199726), except the Goat serum step was not required. Sections known to stain positively were included in each batch and negative controls were prepared by replacing the primary antibody with DAKO antibody diluent. Slides were manually washed in tap water before being rinsed in graded concentrations of alcohol, with a final rinse in Xylene. Glass coverslips were applied. Images were acquired using a NIKON light microscope and a SPOT Insight QE digit camera.

### *In vivo* studies

#### Animal breeding and maintenance

All animal procedures were approved by the University of Dundee Ethical Review Process and were performed in accordance with UK Home Office regulations following PPL70/8579 under the Animals (Scientific Procedures) Act 1986. *Npat* heterozygote (KO first allele reporter-tagged insertion with conditional potential) mice on C57Bl/6Ntac background were purchased from the International Mouse Phenotyping Consortium (IMPC). Both WT and heterozygote *Npat* mice were generated by crossing *Npat* tm1b(EUCOMM)Wtsi males and females. Mice were maintained on RM1 diet (Special Diet Services RM1 pelleted 801151) until they entered the study, and were group-housed, 4 per cage, in the local Animal Resource Unit with *ad libitum* access to food and water under a 12-hour dark/light cycle.

#### Study groups

High fat diet (HFD) (RM AFE 45% fat; 20% protein, 35% carbohydrate) and HFD + 1800ppm Metformin were purchased from Special Diet Services (product codes 824053HFD and 820269 respectively). Male *Npat* heterozygous mice and WT littermate mice were randomly assigned to one of 4 study groups (n = 12–14) at age 8 weeks: WT/HFD, HET/HFD, WT HFD + Metformin (1800ppm), HET HFD + Metformin. Following a short initial acclimatisation period of 50% HFD and 50% RM1 diet, all 4 animal groups were placed on HFD for 23 weeks. Animals were weighed weekly at a consistent time on the same day of each week using electronic scales.

#### Oral glucose tolerance tests (OGTT)

OGTT were performed at weeks 6, 10 and 19 of the study. Animals were weighed at the start of the day and fasted for 4 hr prior to recording basal blood glucose using Bayer Contour^®^ Glucose Meters and strips, by tail vein blood sampling. Animals received an oral 50 mg glucose bolus shortly after measuring baseline glycaemia. Blood glucose was monitored at 0, 15, 30, 45, 60, 90 and 120 min time points. Animals were left unrestrained in their home cages during the procedure.

#### EchoMRI data

Body composition data was obtained using the EchoMRI^™^ 4in1, in line with the protocol provided by the manufacturer (http://www.echomri.com/Body_Composition_4_in_1.aspx) at weeks 0, 10 and 21.

#### Calorimetric measurements (energy expenditure, activity and RER)

Whole body metabolism of all animals was measured in the Comprehensive Lab Animal Monitoring System, CLAMS/Oxymax Activity monitoring system, (Columbus Instruments International©, 2017) with software version 999 from week 23. Animals were acclimatised to single housing at week 22. A 12:12 dark:light cycle was maintained and their access to water and respective diet in power were provided ad libitum. Locomotor activity was measured by beam breaks in the X and Y plane while Z recordings (rearing and jumping) were not included in the analysis. Food intake, activity, heat and respiratory exchange ratio were recorded over the subsequent 48hr.

#### Tissue collection

Schedule 1 was performed on all mice following a recovery of 2 weeks from the CLAMS system. Mice were fasted overnight then half were refed for 1 hr prior to tissue harvest, with the rest sacrificed fasted. Animals were killed by cervical dislocation and confirmation of death was by severing the femoral artery. Tissue was snap frozen in liquid nitrogen and stored at -80°C. Hepatocytes were isolated from a total of 11 WT C57Bl6 animals (male and female mix) from the breeding colony.

### Statistics

Statistical analyses of the biochemical and cellular studies were performed using one-way ANOVA with post hoc Tukey-Kramer test to accommodate for multiple comparisons or Student’s T-test for two groups comparisons, as appropriate. Significance was set at p<0.05. All *in vivo* data are presented as means ± S.E.M. A repeated measures ANOVA was performed with genotype and drug as between subject factors. Statistical analyses were performed using Microsoft Excel, GraphPad Instat 3 and GraphPad Prism 5 software.

## Results

### Development of an NPAT detection assay and genetically modified NPAT cell lines

**Fig 1:**
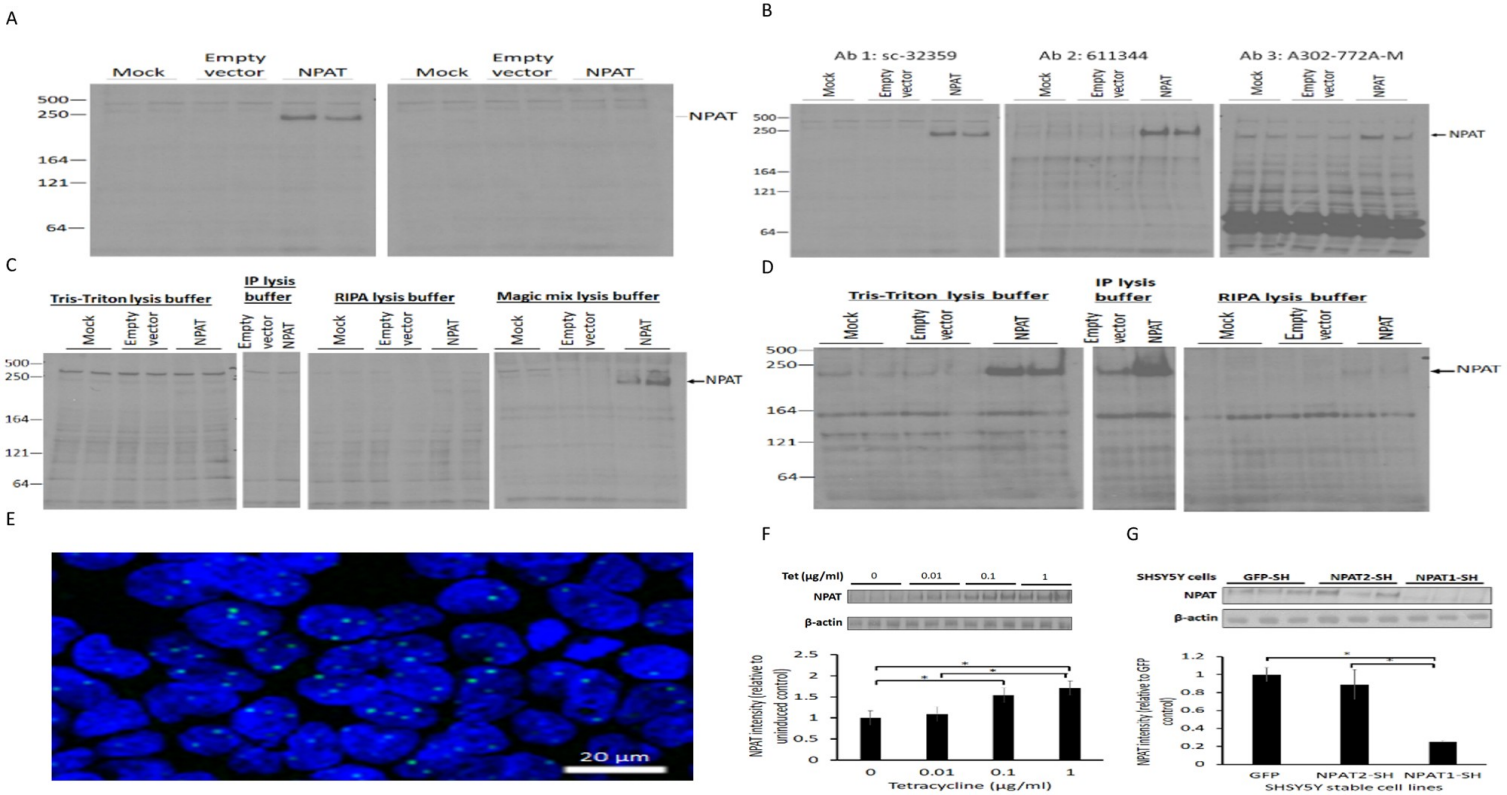
NPAT detection assay and NPAT cell line development. (A-B) HEK293 cells were transfected with NPAT plasmids for 48 hr prior to cell lysis in Urea lysis buffer. NPAT protein was probed by Western blotting with (A) Ab sc32359 (left panel) or Ab sc32359 plus blocking peptide (right panel), and (B) Ab sc32359 (left panel), Ab 611344 (middle panel) or Ab A30-772A-M (right panel). (C) Soluble cell lysates were prepared in Tris-Triton lysis buffer, IP lysis buffer, RIPA lysis buffer or Urea lysis buffer, and probed with Ab sc32359. (D) Insoluble pellets from C were further lysed in Urea lysis buffer and Western blots probed with same antibodies. (E) Immunofluorescence visualization of recombinant NPAT in HEK293 cells, detected with Ab611344. (F) Stably transfected 293 cells were cultured for 24 hr in the presence of tetracycline (Tet) as indicated. NPAT protein was detected in triplicate samples on Western blots with Ab sc32359 and quantified by densitometry (lower panel). (G) Cells expressing GFP-SH (control), NPAT1-SH or NPAT2-SH (shRNA for NPAT) were lysed and NPAT protein detected on Western blots using Ab sc32359. NPAT was quantified by densitometry (lower panel). Data are show as mean ± SEM, n = 3. One-way ANOVA with post hoc Tukey-Kramer multiple comparisons test, *p<0.05.

We tested commercially available NPAT antibodies for sensitivity and specificity using Western blotting. Antibody sc32359 (raised against an epitope near the C-terminus of human NPAT) recognized a protein band at ca 220 kDa in a HEK293 cell lysate (transfected with the NPAT plasmid) (Fig 1A). This band was not detected in lysates prepared from mock transfected cells or cells transfected with empty vector. We further confirmed the specificity of the antibody by loss of the recombinant NPAT signal with a binding site blocking peptide. The difference in apparent molecular mass of the predicted (160 kDa) and the detected NPAT (220 kDa) may indicate post-translational modification. We tested the specificity of two additional NPAT antibodies, 611344 which is specific to human NPAT a.a. 681–803, and A302-772A-M which was raised against human NPAT a.a. 1377–1427. Antibody 611344 produced a similar profile in lysates from cells transfected with human NPAT as antibody sc32359 (Fig 1B). In contrast, antibody A302-772A-M recognised a range of protein bands in all the lysates, suggesting some off-target interactions under these conditions (Fig 1B).

We repeated the analysis of these NPAT antibodies with alternative buffer systems. Over expressed NPAT protein was not detected in cell lysates prepared with the IP lysis buffer or RIPA buffer. However, strong signals were detected in the lysates prepared with Urea lysis buffer (Fig 1C). In addition, NPAT was detected in the pellet fraction of the lysates prepared with the Tris-Triton and IP lysis buffers, indicating that a large fraction of the NPAT protein is insoluble (Fig 1D). Although the over-expressed NPAT was detectable in the lysates prepared with the Urea lysis buffer, endogenous NPAT was detected more strongly after its enrichment in the pellet of the lysates prepared with the IP lysis buffer (Fig 1D). This extraction profile implies that NPAT is mostly associated with the chromatin and the nuclear matrix.

We next investigated the utility of the antibodies for detecting endogenous NPAT and over-expressed NPAT in HEK293 cells by immunofluorescence. We detected NPAT as foci in the nucleus using antibody 611344 only (Fig 1E). This localisation is consistent with the profile of NPAT by Western blotting analysis.

Next, we generated tetracycline-inducible stable cell lines over- or under-expressing NPAT to investigate the impact of NPAT expression level on the cellular response to metformin. NPAT expression was 1.5-fold and 1.7-fold higher in the cells induced for 24 hr with 0.1 μg/ml or 1 μg/ml tetracycline, respectively, compared to those without induction (Fig 1F). Based on these results, 0.1 μg/ml of tetracycline was used in the subsequent experiments with the stable HEK293 cells. We also generated two stable SHSY5Y cell lines carrying lentiviral vectors containing shRNA constructs targeting different regions of the NPAT gene. One of the clones (NPAT1-SH, clone 141527) shows a decrease in the expression of NPAT of 75% compared to the control (GFP-SH) (Fig 1G).

### Characterization of NPAT and ATM expression in cells and mouse tissues

**Fig 2:**
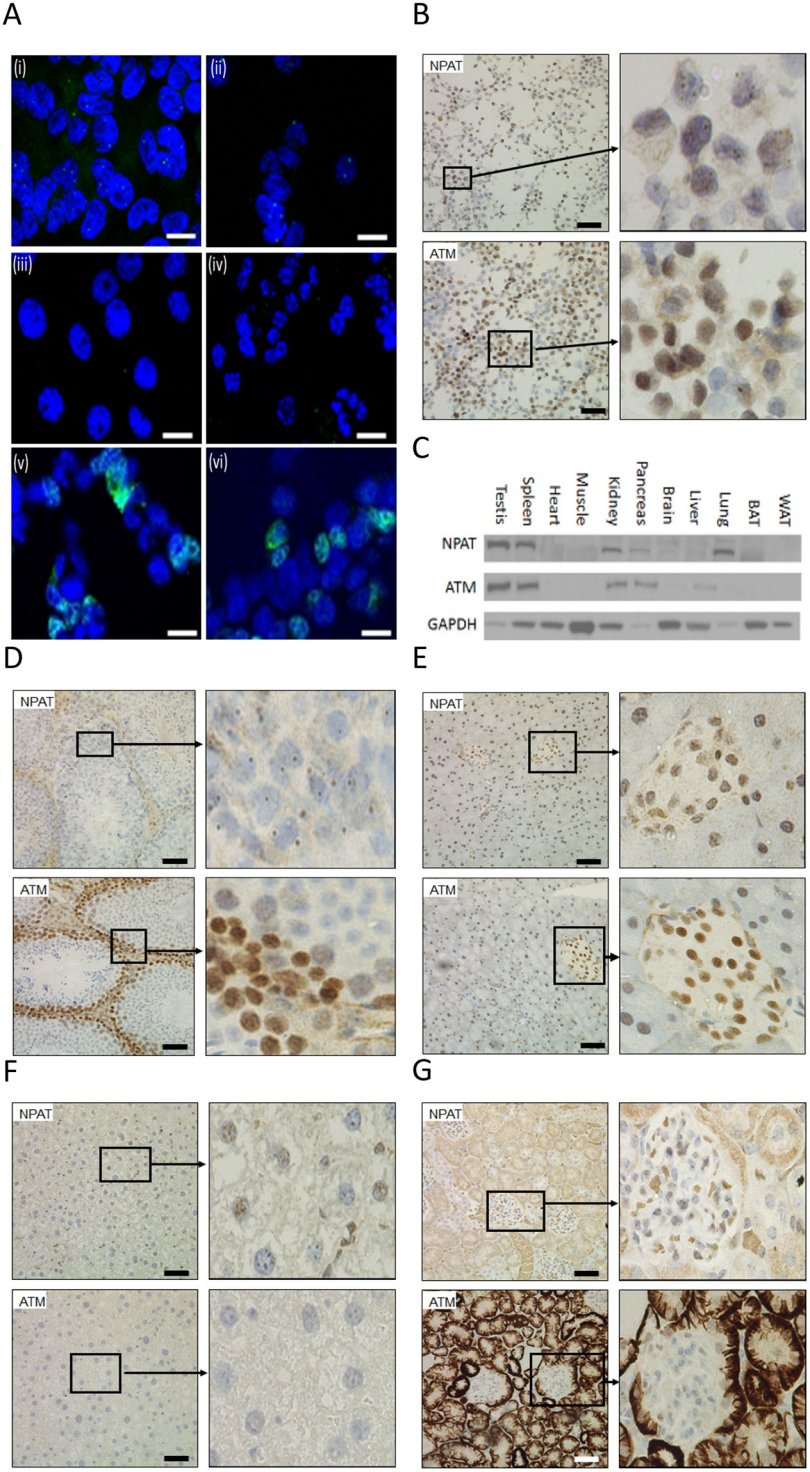
NPAT and ATM expression in cell lines and mouse tissues. (A) Immunofluorescence staining (green) of endogenous NPAT in HEK293 (Ai), HepG2 (Aii), H4IIe (Aiii) cells and primary mouse hepatocytes (Aiv); Immunofluorescence staining of ATM in HEK293 cells transfected with plasmids carrying either wild-type ATM (Av) or ATM with a kinase dead mutation (Avi). Bar: 20 μm. Nuclei stained blue. (B) Immunohistochemical staining of NPAT and ATM in HEK293 cells transfected with plasmids carrying either wild-type NPAT or ATM. Bar: 50 μm. (C) Western blotting analysis of NPAT and ATM in mouse tissues. Immunohistochemical staining of NPAT and ATM in mouse testis (D), pancreas (E), liver (F) and kidney (G); Bar: 50 μm.

There is limited information on the subcellular localisation of ATM and NPAT protein, and the evidence of functional interaction between these gene products is only at the level of gene expression (due to a shared gene promoter element and the binding of each protein to the gene promoters). Therefore, we investigated whether NPAT and ATM were co-localised in cell lines and tissues. Endogenous NPAT was detectable (green) in HEK293 (Fig 2Ai) and HepG2 cells (Fig 2Aii) but not in H4IIe cells (Fig 2Aiii) or mouse primary hepatocytes (Fig 2Aiv). NPAT appears as discrete foci in the nucleus (DAPI-blue, 1–4 foci/nucleus). Endogenous ATM was undetectable by immunofluorescence, therefore we transiently transfected HEK293 cells with two plasmids carrying either the wild-type ATM or the ATM with a kinase dead mutation. Recombinant ATM is mostly localized in the nucleus, with some cytoplasmic staining. The patterns of the ATM staining are similar for both the wild-type (Fig 2Av) and the mutant ATM (Fig 2Avi), indicating that the kinase dead mutation does not affect the localization of ATM protein.

Immunocytochemistry of transfected HEK293 cells also detected NPAT in the nucleus as discrete brown foci with minimal background staining (Fig 2B, upper panels). Strong ATM brown staining is restricted to the nucleus but in contrast to NPAT weak staining is also seen in cytoplasm (Fig 2B, bottom panels).

We next analyzed the expression of NPAT and ATM protein in a range of mouse tissues by Western blots. NPAT and ATM proteins are detectable in testis, spleen, kidney, pancreas and lung tissues, with various intensities (Fig 2C and Table 1). This pattern was unexpected and raised the possibility that NPAT and ATM may influence glucose metabolism/metformin action through pancreatic or kidney actions rather than liver or intestine as expected. IH analysis of mouse tissues confirmed these data with strongest staining in testis (Fig 2D). As seen in the upper panels, the immunostaining of NPAT appears in germ cells, i.e. spermatogonium and spermatocytes. As the cells mature, the staining is lost. NPAT is again localised in the nucleus as discrete foci. Interestingly, immunostaining of ATM is also found in the germ cells, with staining lost as the cells mature. ATM protein staining in the nucleus was much more diffuse than NPAT. In the pancreas, NPAT and ATM are detected primarily in a subset of cells in the islets of Langerhans but also in surrounding pancreatic acinar cells (Fig 2E). In liver, immunostaining of NPAT is in a subset of hepatocytes and probably Kupffer cells, again as discrete nuclear foci (Fig 2F, upper panels). ATM is undetectable in the liver (Fig 2F, bottom panels). Very intense cytoplasmic staining of renal tubules and Bowman’s capsule in the kidney is found for both NPAT and ATM (Fig 2G). This pattern requires confirming with alternative ATM and NPAT antibodies, when available.

**Table 1:** Summary of detection of endogenous NPAT and ATM in mouse tissues.

Tissues	NPAT		ATM	
	IH	WB	IH	WB
Testis	+++	+++	+++	+++
Spleen	NA	+++	NA	+++
Heart	NA	ND	NA	ND
Muscle	NA	ND	NA	ND
Kidney	+++	++	+++	++
Pancreas	++	+	++	+
Brain	NA	ND	NA	ND
Liver	+	ND	ND	+
Lung	NA	++	NA	ND
Brown adipose tissue (BAT)	NA	ND	NA	ND
Write adipose tissue (WAT)	NA	ND	NA	ND

IH: immunohistochemistry; WB: Western blotting; +++: strong signal; ++: intermediate signal; +: weak signal; NA: not applicable; ND: not detectable.

### NPAT and ATM are not mutually regulated

**Fig 3:**
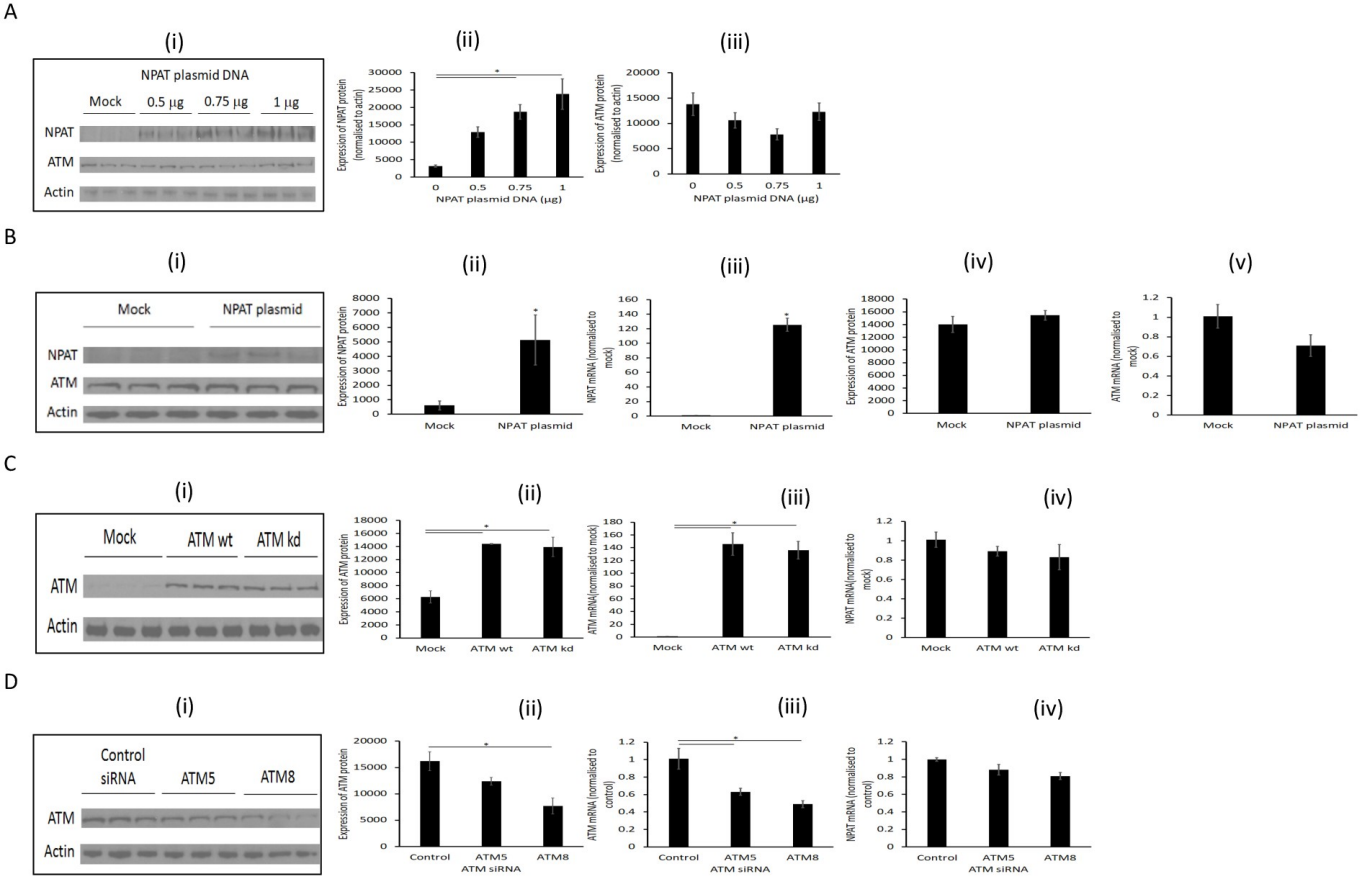
Changing NPAT expression in HEK293 cells had no effect on the expression of ATM and *vice versa*. (A) HEK293 cells were transfected with NPAT plasmid for 24 hr as indicated. The expression of NPAT and ATM was detected by Western blotting (Ai) and quantified by densitometry (Aii and iii). (B) HEK293 cells were transfected with 1 μg of NPAT plasmid for 24 hr. NPAT and ATM were detected by Western blotting (Bi) and quantified by densitometry (Bii & Biv). NPAT and ATM mRNA were analysed by real-time PCR using Taqman assay (Biii & Bv). (C) HEK293 cells were transfected with plasmids encoding ATM wild-type (ATM wt) or ATM with kinase dead mutation (ATM kd) for 24 hr. ATM was detected on Western blots (Ci) and quantified by densitometry (Cii). ATM and NPAT mRNA were analysed by real-time PCR using Taqman assay (Ciii & Civ). (D) HEK293 cells were transfected with ATM siRNA, ATM5 and ATM8 for 48 hr. ATM was detected by Western blotting (Di) and quantified by densitometry (Dii). ATM and NPAT mRNA were analysed by real-time PCR using Taqman assay (Diii & Div). Data are mean ± SEM, n = 3, and analysed by One-way ANOVA with post hoc Tukey-Kramer multiple comparisons test (A, C and D), or unpaired Student T-test, *p<0.05.

The *NPAT* and *ATM* genes are physically linked, and previous work proposed that they were functionally associated, with NPAT modifying ATM expression. However, we found that transfection of increasing amounts of NPAT expression plasmids into HEK293 cells increased the amount of NPAT protein (Fig 3Ai and 3Aii), without significantly altering ATM protein levels (Fig 3Aiii). Similarly, transfection of HEK 293 cells with 1 μg of NPAT expression plasmid (Fig 3Bi) generated a 5-fold increase in NPAT protein (Fig 3Bii), from a 100-fold increase in NPAT mRNA (Fig 3Biii). Again, this had no significant impact on ATM protein (Fig 3Biv) or mRNA (Fig 3Bv). The results indicate that simple overexpression of NPAT does not affect endogenous ATM gene expression in HEK293 cells.

For completion, we overexpressed ATM in these cells (Fig 3Ci). HEK293 cells were transfected with an expression plasmid for either wild-type ATM or ATM with a kinase dead (KD) mutation. The transfections resulted in significant increases in ATM protein (Fig 3Cii) and mRNA (Fig 3Ciii), with no significant change in NPAT mRNA (Fig 3Civ). Hence, simple overexpression of ATM has no effect on the expression of endogenous NPAT in these cells. Next, we transfected HEK 293 cells with siRNA oligos, ATM5 and ATM8 for 48 hr (Fig 3Di). ATM8 resulted in a significant reduction of ATM protein compared to the control, while ATM5 had a lesser effect (Fig 3Dii). ATM mRNA was reduced in line with the impact on ATM protein (Fig 3Diii). In contrast, these changes in ATM expression did not alter NPAT mRNA (Fig 3Div). Hence, these factors do not appear to influence the expression of each other, at least in this timeframe.

### Changing NPAT expression does not affect cell sensitivity to biguanides

**Fig 4:**
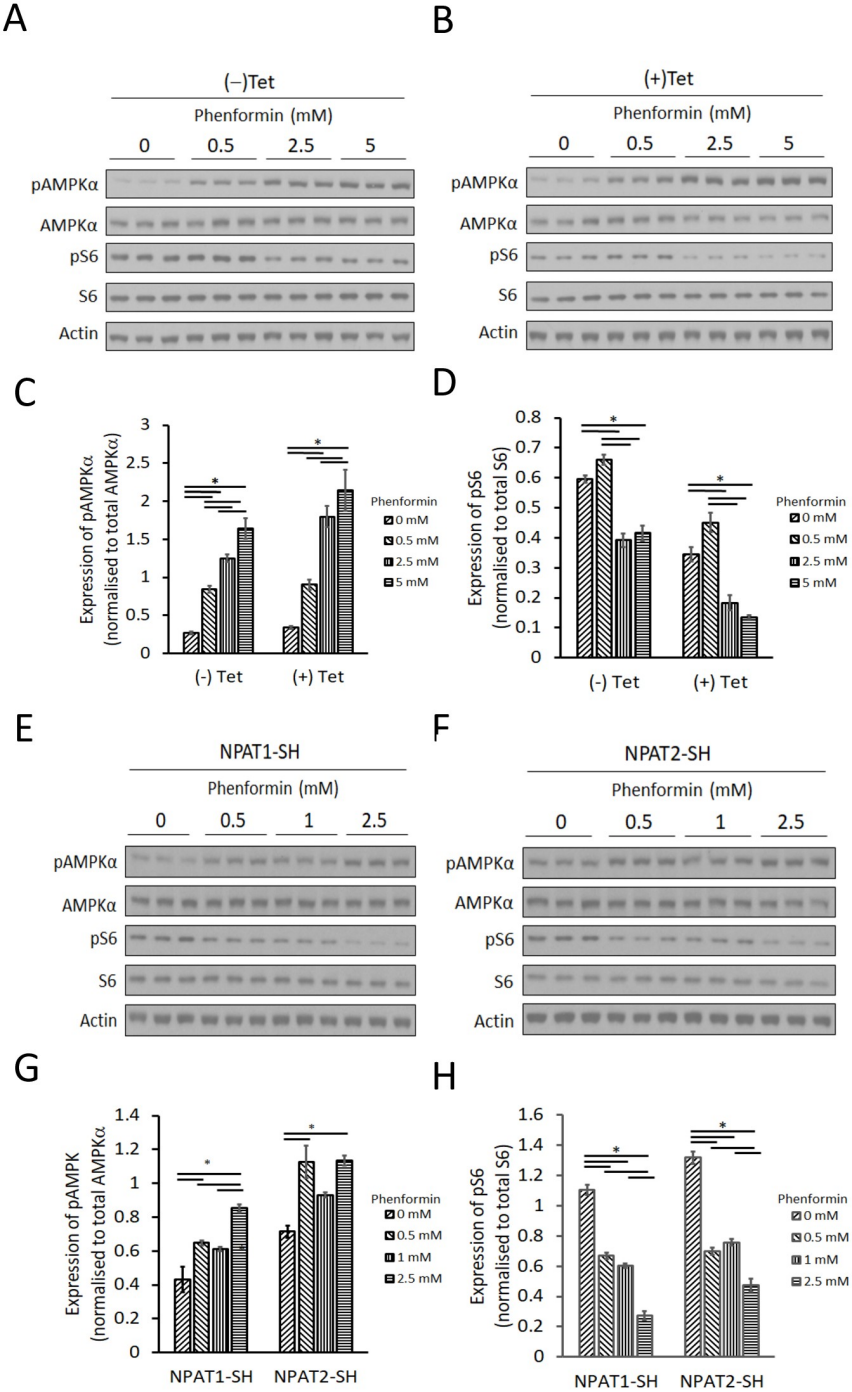
Changing NPAT expression has no significant effect on cell signalling induced by phenformin. (A & B) Western blotting analysis of phospho-AMPKα (P-Thr172) (pAMPKα), AMPKα, phospho-S6 (P-Ser240/P-Ser244) (pS6), S6 and actin in cell lysates from HEK293 stably transfected with inducible *NPAT* (See Fig 1F). Cells were treated with phenformin in the absence (-Tet) (A) or presence (+Tet) (B) of tetracycline (0.1 μg/ml) for 6 hr to induce NPAT production. Western blotting data was quantified by densitometry (C & D). Western blotting analysis of pAMPKα, AMPKα, pS6, S6 and actin (E & F), in cell lysates from NPAT1-SH and NPAT2-SH cells (NPAT knockdown-see Fig 1G). Cells were treated with phenformin for 6 hr, and immunoblots quantified by densitometry (G & H). Data are mean ± SEM, n = 3. One-way ANOVA with post hoc Tukey-Kramer multiple comparisons test was used to calculate the significant difference between groups. *p<0.05.

The *NPAT* gene lies within a genetic locus that associates with metformin response in humans with type 2 diabetes [11]. This implies that changes in NPAT expression (or activity) moderate metformin control of glucose homeostasis, and this could be due to changes in metformin sensitivity at the level of cell signaling. We analyzed two of the best studied cellular pathways regulated by metformin, namely the AMPK and S6K pathways. However, HEK 293 and SHSY5Y cells responded relatively poorly to acute metformin challenge, even at concentrations as high as 5 mM. In contrast, the more potent biguanide, phenformin, generated a robust acute response in these cells. In the absence of NPAT induction, treating HEK 293 cells with phenformin for 6 hr (-Tet) generated a dose-dependent increase of pAMPK, and a dose-dependent decrease of pS6 (Fig 4A and 4C). Inducing NPAT production with tetracycline (+Tet, see Fig 1F), did not alter the phenformin sensitivity of these two pathways (Fig 4B and 4D). Hence, over-expression of NPAT in these cells didn’t change their sensitivity to acute phenformin challenge (Fig 4A–4D). Treatment of SHSY5Y-NPAT1 (low NPAT level) and SHSY5Y-NPAT2 (higher NPAT level) cells (see Fig 1G for knockdown) with phenformin for 6 hr resulted in a significant increase of pAMPK and a significant decrease of pS6 (Fig 4E–4H). There was no significant difference between these two cell lines suggesting that lowering NPAT (NPAT1-SH) does not change the sensitivity of the cells to biguanides (Fig 4E–4H).

### Phenotypic analysis of *npat*^+/-^ heterozygous mice

**Fig 5:**
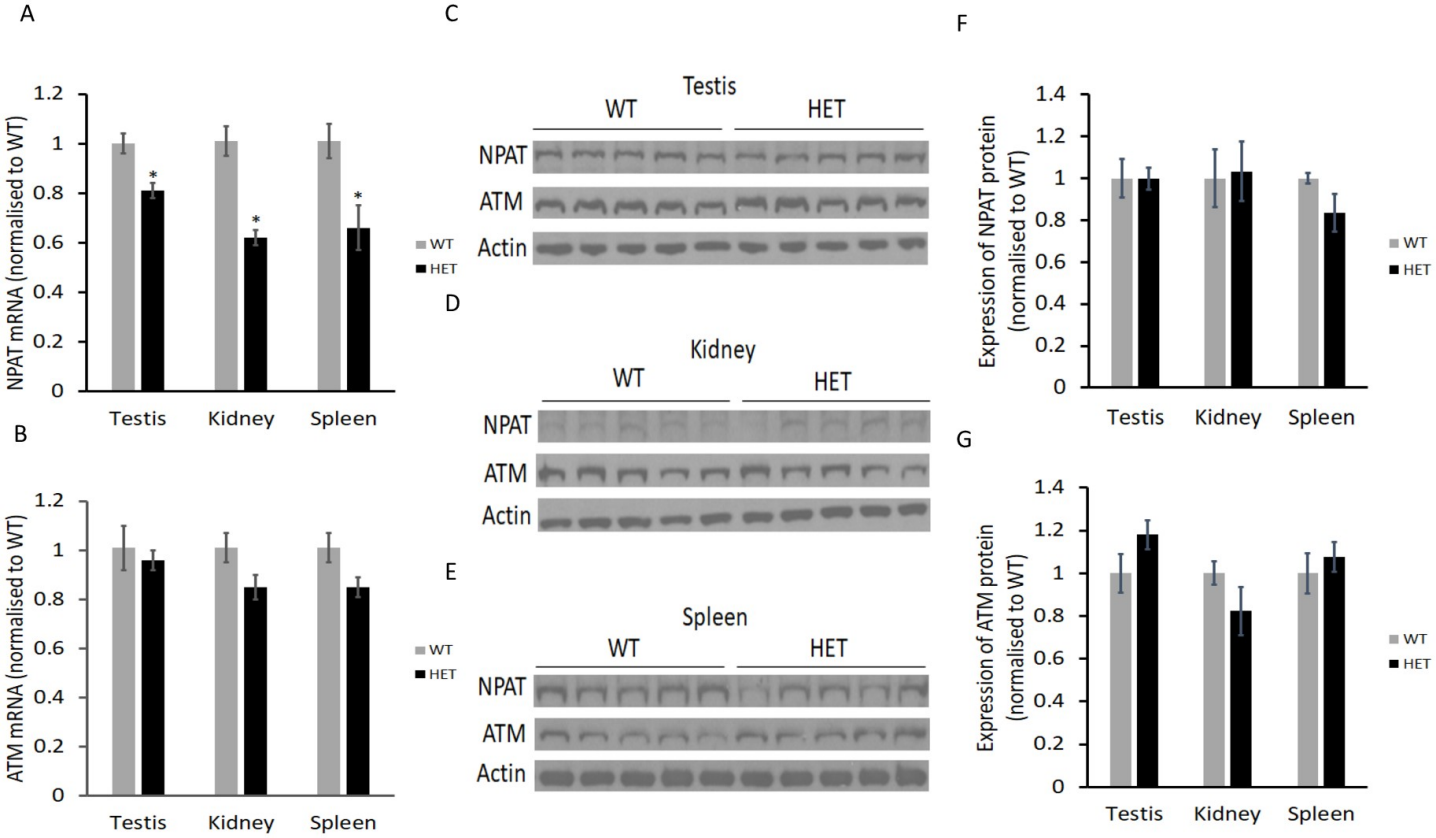
Expression of NPAT and ATM in mouse tissues. (A & B) NPAT and ATM mRNA obtained by real time PCR. RNA was extracted from testis, kidney and spleen tissues of wild-type (WT) and heterozygous (HET) *NPAT*^*+/-*^ mice and mRNA expression levels were calculated relative to the wild-type. (C–E) Detection of NPAT and ATM by Western blotting. Testis, kidney and spleen tissues were collected from wild-type (WT) and heterozygous (HET) *NPAT*^*+/-*^ mice and tissue lysates were prepared in Urea lysis buffer. Cellular proteins (25 μg) were analysed by Western blotting using specific primary antibodies for NPAT, ATM and actin. (F & G) Quantification of NPAT and ATM proteins by densitometry. Protein levels were calculated relative to the wild-type. Data are mean ± SEM, n = 5. Unpaired Student t-test was used to calculate the significant difference between groups. *p<0.05.

We compared the expression of NPAT and ATM in tissues from *npat* heterozygous mice (*npat*^+/-^) and the wild-type littermates. Complete deletion of *npat* is embryonic lethal. Deletion of one allele of *npat* resulted in a reduction of NPAT mRNA by 20–40% in testis, kidney and spleen (Fig 5A). However, the mRNA of ATM was not significantly different in these tissues from *npat* heterozygous mice compared to those from the wild-type littermates (Fig 5B). The reduction of NPAT mRNA in these tissues did not induce significant changes in NPAT or ATM protein levels, at least when measured by Western blotting (Fig 5C–5G). Of course, we cannot rule out that deletion of an *npat* allele reduces NPAT protein expression in other tissues, or that the Western blot technique with the available antibody is not sufficiently sensitive to detect 10–30% changes in NPAT protein.

**Fig 6:**
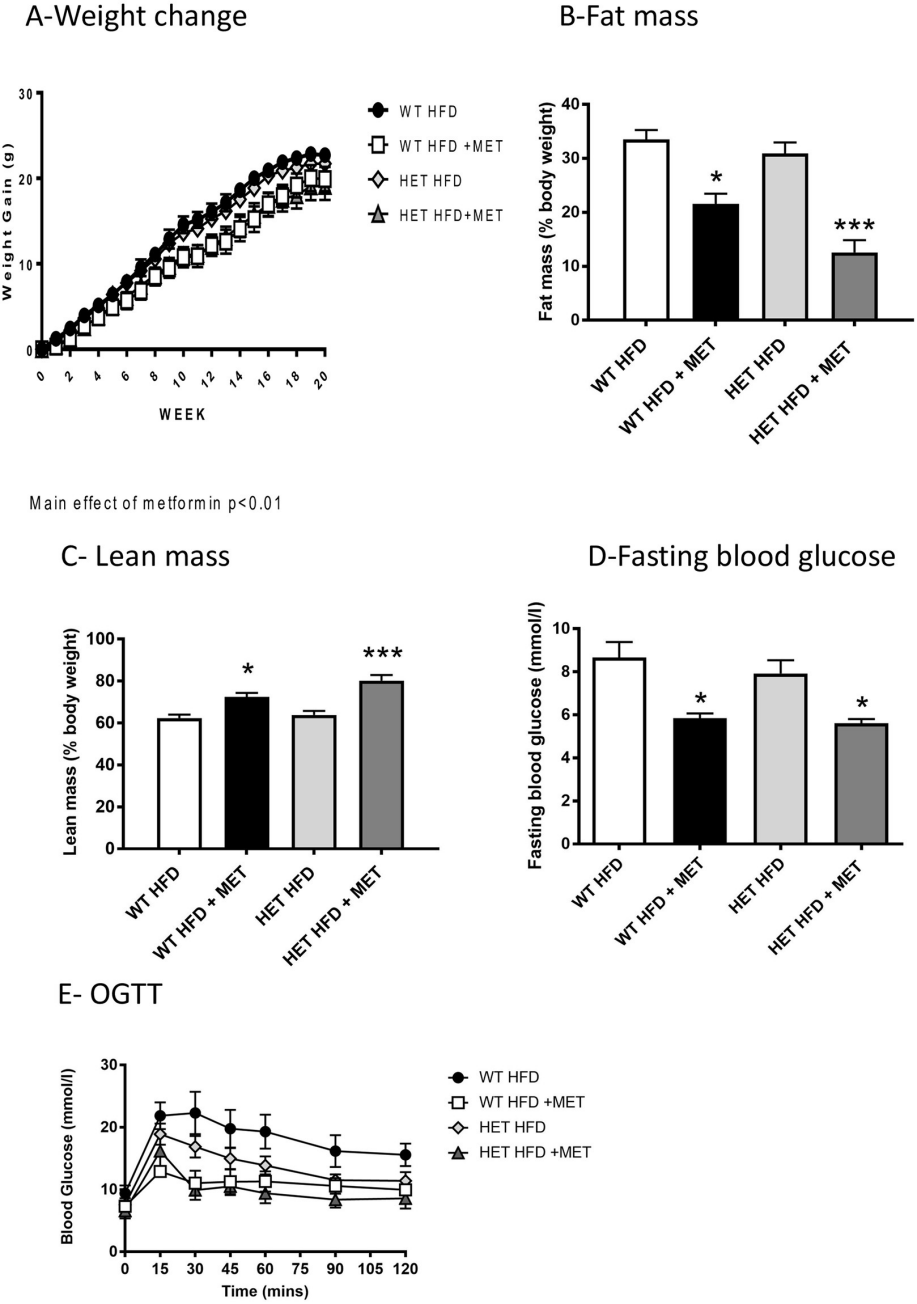
Body composition and metabolic analysis. (A) All animals were weighed weekly throughout the study, data is presented as weight change from study initiation. (B and C) Body composition data was obtained using the EchoMRI^™^ 4in1. Data from week 10 of the study is presented with similar distribution at week 19. (D) Fasting blood glucose was performed at week 20. (E) OGTT was performed at week 19 in all animals.

To investigate if deletion of one allele of *npat* would alter body weight, glucose metabolism or actions of metformin *in vivo*, *npat*^+/-^ mice and their WT littermate controls were fed with a high-fat diet supplemented with metformin and metabolic parameters assessed. This fat feeding model is commonly used to induce insulin resistance and glucose intolerance in the C57Bl6 mice [23]. Metformin treatment significantly reduced weight gain (Fig 6A) in both genotypes (8–12 weeks after treatment initiation), compared to no drug controls. The blunted weight gain was accompanied by a metformin-associated reduction in percentage fat mass (Fig 6B), a concomitant increase in percentage lean mass (Fig 6C) and a lower fasting blood glucose (Fig 6D), in both WT and *npat*^+/-^ mice. Supplementation of the diet with metformin improved glucose clearance following an oral glucose tolerance test (Fig 6E; genotype x drug; p<0.05). However, the action of metformin on OGTT in npat^+/-^ mice was not significant (genotype x drug; p = 0.46), using repeated ANOVA measures. This may be due to the lower levels of glucose accumulation in the blood following the OGTT (Fig 6E), however the repeated ANOVA did not detect a significant genetic influence in the absence of metformin either. It is therefore difficult to interpret the physiological impact of loss of one npat allele using the OGTT. To investigate alterations in whole-body metabolism in more detail indirect calorimetry was performed.

**Fig 7:**
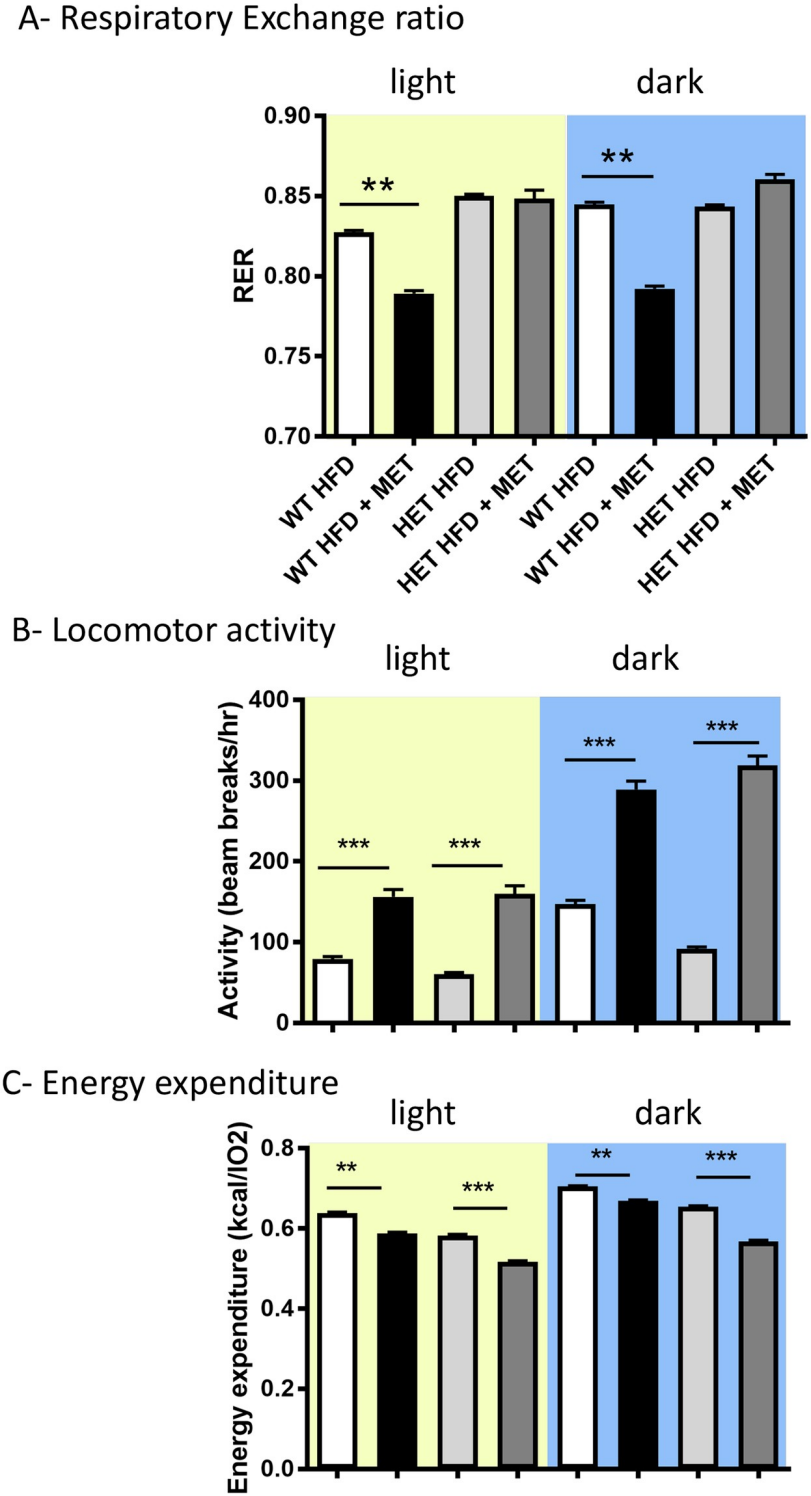
Energy and activity analysis. (A) Respiratory Exchange Ratio (RER), (B) Activity and (C) Energy Expenditure were calculated at end of study (week 22) using the Comprehensive Lab Animal Monitoring System, CLAMS/Oxymax Activity monitoring system. A 12:12 dark:light cycle was maintained with access to water and food ad libitum. Food intake, activity, heat and respiratory exchange ratio were recorded over 48hr.

Most interesting was a significant genotype x drug interaction in the respiratory exchange ratio (RER, (Fig 7A; p<0.01)), with metformin significantly reducing the RER in WT animals during both light and dark phases (p<0.01); however, there was no metformin regulation of the RER in *npat*^+/-^ animals. Meanwhile, all animals were more active in the dark phase (Fig 7B; effect of phase, p<0.01), with metformin-treated groups moving more than those receiving HF diet alone (effect of drug, p<0.001). Surprisingly, the metformin-associated increase in locomotor activity was accompanied by a genotype-independent reduction in energy expenditure in both the light and dark phase (Fig 7C, effect of phase; p<0.05; effect of drug p<0.001).

In summary, the most commonly measured metabolic parameters were not affected by loss of one allele of *npat*. The main exception was with metformin regulation of RER, which was only seen in the wild-type mice. This suggests that a full complement of NPAT is required for metformin to modify fuel utilisation in mice.

## Discussion

GWAS approaches have identified genetic polymorphisms that associate with health phenotypes in a range of human diseases [11]. They have increasingly been used to identify genetic variants that are associated with drug metabolism, drug efficacy and adverse effects [24]. Although huge numbers of SNP-trait associations have been identified, only a small fraction of these associations have subsequently been investigated experimentally [24, 25]. Moving from GWAS to biology can be very challenging, in part due to the vast number of ‘hits’ usually identified, but also as most SNPs identified are not directly associated with coding changes or expression of known gene products. In addition, SNPs are often not conserved in the genome of animal models routinely used in experimental biology (as human SNPs occur more frequently in regions with less evolutionarily conservation [26]). This means that most experimental follow ups have to focus on genes that lie ‘close’ to SNPs of interest, and delete or overexpress those genes [27].

Our GWAS on glycaemic response to metformin in patients with type 2 diabetes identified a strong association with variants at a locus on chromosome 11 containing 7 genes. Following the initial discovery, further studies have established stronger links between the GWAS signal and the response to metformin. In particular, a specific variant in NPAT gene sequence, which results in a coding change L540F, fully explained the GWAS signal suggesting that this was the causal variant. Meanwhile, ataxia-telangiectasia (A-T) is caused by inactivating mutations in ATM, the gene that lies next to NPAT on chromosome 11, and is associated with metabolic dysfunction, leading to insulin resistance, diabetes and fatty liver [20, 21, 28–30]. This association has been described repeatedly since case reports in the 1970s, and latterly in mouse models of A-T [28, 29]. More recently, using oral glucose tolerance tests, we confirmed insulin resistance in an A-T patient group compared to control [30].

Therefore, there are several lines of evidence suggesting that the DNA sequence in the ATM and NPAT gene region influences metformin response and glucose metabolism. In the present study, we focused on the NPAT gene, however previous work had indicated the possibility that the ATM gene product could regulate NPAT, and vice versa. If true, then the SNPs in each gene that associate with metformin response may actually share a common mechanism. However, our data does not support the reciprocal regulation of these gene products. *In vitro* and *in vivo* analysis of systems with modified NPAT production did not regulate ATM transcription, and we have data from ATM deficient mice showing no change in NPAT regulation. Similarly, altering the expression of NPAT in mice had little effect on multiple aspects of glucose metabolism and/or the pharmacological response to metformin. The interesting exception was in control of respiratory exchange ratio (RER).

The RER is the ratio between carbon dioxide produced in metabolism and oxygen consumed. It provides an indication of the fuel that is being oxidised for energy production. An RER of 0.7 indicates fat as the predominant fuel source, while an RER of 1.0 indicates carbohydrate. A value between 0.7 and 1.0 shows that both are being used. We found that metformin reduced the RER in mice on a high fat diet, indicating that it is making the animals metabolise relatively more fat. These data may indicate that an important aspect of metformin action is to enhance dynamic fuel switching (fuel/metabolic flexibility). Indeed, the inability to increase fat metabolism (i.e. blunted fuel switching) is a feature of insulin resistance, and muscle from T2D patients exhibits decreased fat oxidation capacity. This reduction of RER by metformin has been previously reported in healthy humans [31], and in people with type 2 diabetes [32]. Indeed, reduction in RER by metformin has been postulated to contribute to the benefits of metformin seen in people with type 2 diabetes [33, 34], despite the limited experimental evidence to demonstrate a reduced metabolic flexibility in diabetes. The small reduction in whole body NPAT production generated by deletion of one allele of *npat*, completely removed this action of metformin in our fat-fed animals. Humans with a SNP in *NPAT* that reduces its expression may not have access to this beneficial action of metformin. As such, we propose that this mechanism contributes to the association of the *NPAT* gene sequence to metformin response.

Additional unusual physiochemical properties are observed in individuals with ataxia- telangiectasia, such as high levels of plasma alpha-fetoprotein and an increased sensitivity to ionising radiation. Radiation treatment is known to alter metabolic status, mostly through associated DNA-damage, and it is possible that the increased radiosensitivity in A-T patients is mediated by the altered ATM and/or NPAT regulation of glucose homeostasis. Equally, some of the impact of ATM on glucose homeostasis may be related to the role ATM has on DNA repair mechanisms [35].

To our knowledge, this is the first gene product, identified by GWAS, that has been linked to metformin control of metabolic flexibility. However, it will require a human study, recruiting by genotype, and assessing metformin regulation of RER, to establish a clear dependence of this metformin action on *NPAT* gene sequence. The data presented here provides an ethical and scientific justification for such a study, while providing methodological developments to allow NPAT assessment. Improved understanding of this mechanism has the potential to generate alternative strategies to modify metabolic flexibility in those patients with the metformin resistant NPAT genotype. Clearly, despite the loss of this action of metformin we did not detect differences in metformin regulation of fat content or the majority of glucose homeostasis in these mice. This may suggest NPAT-independent actions of metformin that can overcome this function of NPAT in the mouse, or under the treatment conditions employed in the fat feeding model. For example, the metformin is given with the diet, and hence this is really a ‘prevention’ design, while the majority (if not all) individuals with diabetes are given metformin potentially years after the initiation of insulin resistance and obesity. Similarly, the model is really one of impaired fasting glucose, or ‘pre-diabetes’ as mice are resistant to developing diabetes in response to poor diet. Hence, establishing the clinical importance of this novel observation, placing NPAT between metformin and control of RER, will have to await human studies in people with type 2 diabetes.

### Limitations of our study

While we have tried to use distinct approaches to address the pharmacogenomic effect of altering NPAT we have only studied outcomes in cells and in mice, where genetic manipulation is more feasible. In addition, we have focused on a single model of obesity-related diabetes development, and the mice do not fully develop diabetes. Finally, we have used a dose of metformin related to that used in humans, which is lower than doses normally used in mouse studies, and as such some of our drug responses are small, making identification of genetic influence more challenging. That said, the work provides evidence for an impact of *npat* on RER, and as such supports the case to perform a trial using recruitment by genotype and RER as a primary outcome.

In summary, we have developed new tools and methodology for the study of NPAT, a gene associated with metformin efficacy in humans. We provide evidence that ATM does not regulate NPAT expression and NPAT does not directly regulate ATM expression. The biological mechanism that underpins the genetic association with metformin efficacy may include NPAT influencing metformin control of metabolic flexibility. This data now justifies a human study to establish the influence of NPAT genotype on metformin regulation of RER.

## Supporting information

S1 Fig Original data for all images shown. (PDF)

S1 Table Source of antibodies. (DOCX)

S2 Table Source of plasmids, vectors and transduction particles. (DOCX)

S3 Table Source of oligos. (DOCX)

## References

[1] Zhou G, Myers R, Li Y, Chen Y, Shen X, Fenyk-Melody J, et al. Role of AMP-activated protein kinase in mechanism of metformin action. J Clin Invest. 2001;108(8):1167–74. doi: 10.1172/JCI13505 11602624 PMC209533

[2] Rena G, Pearson ER, Sakamoto K. Molecular mechanism of action of metformin: old or new insights? Diabetologia. 2013;56(9):1898–906. doi: 10.1007/s00125-013-2991-0 23835523 PMC3737434

[3] Foretz M, Guigas B, Bertrand L, Pollak M, Viollet B. Metformin: from mechanisms of action to therapies. Cell Metab. 2014;20(6):953–66. doi: 10.1016/j.cmet.2014.09.018 25456737

[4] Owen MR, Doran E, Halestrap AP. Evidence that metformin exerts its anti-diabetic effects through inhibition of complex 1 of the mitochondrial respiratory chain. Biochem J. 2000;348 Pt 3:607–14. 10839993 PMC1221104

[5] Kalender A, Selvaraj A, Kim SY, Gulati P, Brule S, Viollet B, et al. Metformin, independent of AMPK, inhibits mTORC1 in a rag GTPase-dependent manner. Cell Metab. 2010;11(5):390–401. doi: 10.1016/j.cmet.2010.03.014 20444419 PMC3081779

[6] Kickstein E, Krauss S, Thornhill P, Rutschow D, Zeller R, Sharkey J, et al. Biguanide metformin acts on tau phosphorylation via mTOR/protein phosphatase 2A (PP2A) signaling. Proc Natl Acad Sci U S A. 2010;107(50):21830–5. doi: 10.1073/pnas.0912793107 21098287 PMC3003072

[7] Ben Sahra I, Regazzetti C, Robert G, Laurent K, Le Marchand-Brustel Y, Auberger P, et al. Metformin, independent of AMPK, induces mTOR inhibition and cell-cycle arrest through REDD1. Cancer Res. 2011;71(13):4366–72. doi: 10.1158/0008-5472.CAN-10-1769 21540236

[8] Vazquez-Martin A, Oliveras-Ferraros C, Cufi S, Martin-Castillo B, Menendez JA. Metformin activates an ataxia telangiectasia mutated (ATM)/Chk2-regulated DNA damage-like response. Cell Cycle. 2011;10(9):1499–501. doi: 10.4161/cc.10.9.15423 21566461

[9] Gong L, Goswami S, Giacomini KM, Altman RB, Klein TE. Metformin pathways: pharmacokinetics and pharmacodynamics. Pharmacogenet Genomics. 2012;22(11):820–7. doi: 10.1097/FPC.0b013e3283559b22 22722338 PMC3651676

[10] Pawlyk AC, Giacomini KM, McKeon C, Shuldiner AR, Florez JC. Metformin pharmacogenomics: current status and future directions. Diabetes. 2014;63(8):2590–9. doi: 10.2337/db13-1367 25060887 PMC4113063

[11] Zhou K, Bellenguez C, Spencer CC, Bennett AJ, Coleman RL, Tavendale R, et al. Common variants near ATM are associated with glycemic response to metformin in type 2 diabetes. Nat Genet. 2011;43(2):117–20. doi: 10.1038/ng.735 21186350 PMC3030919

[12] Byrd PJ, Cooper PR, Stankovic T, Kullar HS, Watts GD, Robinson PJ, et al. A gene transcribed from the bidirectional ATM promoter coding for a serine rich protein: amino acid sequence, structure and expression studies. Hum Mol Genet. 1996;5(11):1785–91. doi: 10.1093/hmg/5.11.1785 8923007

[13] Imai T, Yamauchi M, Seki N, Sugawara T, Saito T, Matsuda Y, et al. Identification and characterization of a new gene physically linked to the ATM gene. Genome Res. 1996;6(5):439–47. doi: 10.1101/gr.6.5.439 8743993

[14] Saarinen S, Aavikko M, Aittomaki K, Launonen V, Lehtonen R, Franssila K, et al. Exome sequencing reveals germline NPAT mutation as a candidate risk factor for Hodgkin lymphoma. Blood. 2011;118(3):493–8. doi: 10.1182/blood-2011-03-341560 21562039

[15] Medina R, van der Deen M, Miele-Chamberland A, Xie RL, van Wijnen AJ, Stein JL, et al. The HiNF-P/p220NPAT cell cycle signaling pathway controls nonhistone target genes. Cancer Res. 2007;67(21):10334–42. doi: 10.1158/0008-5472.CAN-07-1560 17974976

[16] DeRan M, Pulvino M, Greene E, Su C, Zhao J. Transcriptional activation of histone genes requires NPAT-dependent recruitment of TRRAP-Tip60 complex to histone promoters during the G1/S phase transition. Mol Cell Biol. 2008;28(1):435–47. doi: 10.1128/MCB.00607-07 17967892 PMC2223310

[17] Savitsky K, Bar-Shira A, Gilad S, Rotman G, Ziv Y, Vanagaite L, et al. A single ataxia telangiectasia gene with a product similar to PI-3 kinase. Science. 1995;268(5218):1749–53. doi: 10.1126/science.7792600 7792600

[18] Dahl ES, Aird KM. Ataxia-Telangiectasia Mutated Modulation of Carbon Metabolism in Cancer. Front Oncol. 2017;7:291. doi: 10.3389/fonc.2017.00291 29238697 PMC5712564

[19] Zaki-Dizaji M, Akrami SM, Abolhassani H, Rezaei N, Aghamohammadi A. Ataxia telangiectasia syndrome: moonlighting ATM. Expert Rev Clin Immunol. 2017;13(12):1155–72. doi: 10.1080/1744666X.2017.1392856 29034753

[20] Schalch DS, McFarlin DE, Barlow MH. An unusual form of diabetes mellitus in ataxia telangiectasia. N Engl J Med. 1970;282(25):1396–402. doi: 10.1056/NEJM197006182822503 4192270

[21] Bar RS, Levis WR, Rechler MM, Harrison LC, Siebert C, Podskalny J, et al. Extreme insulin resistance in ataxia telangiectasia: defect in affinity of insulin receptors. N Engl J Med. 1978;298(21):1164–71. doi: 10.1056/NEJM197805252982103 651946

[22] Logie L, Harthill J, Patel K, Bacon S, Hamilton DL, Macrae K, et al. Cellular responses to the metal-binding properties of metformin. Diabetes. 2012;61(6):1423–33. doi: 10.2337/db11-0961 22492524 PMC3357267

[23] Kleinert M, Clemmensen C, Hofmann SM, Moore MC, Renner S, Woods SC, et al. Animal models of obesity and diabetes mellitus. Nat Rev Endocrinol. 2018;14(3):140–62. doi: 10.1038/nrendo.2017.161 29348476

[24] Visscher PM, Wray NR, Zhang Q, Sklar P, McCarthy MI, Brown MA, et al. 10 Years of GWAS Discovery: Biology, Function, and Translation. Am J Hum Genet. 2017;101(1):5–22. doi: 10.1016/j.ajhg.2017.06.005 28686856 PMC5501872

[25] Bush WS, Moore JH. Chapter 11: Genome-wide association studies. PLoS Comput Biol. 2012;8(12):e1002822. doi: 10.1371/journal.pcbi.1002822 23300413 PMC3531285

[26] Castle JC. SNPs occur in regions with less genomic sequence conservation. PLoS One. 2011;6(6):e20660. doi: 10.1371/journal.pone.0020660 21674007 PMC3108954

[27] Petrie JR, Pearson ER, Sutherland C. Implications of genome wide association studies for the understanding of type 2 diabetes pathophysiology. Biochem Pharmacol. 2010. doi: 10.1016/j.bcp.2010.11.010 21111713

[28] Schneider JG, Finck BN, Ren J, Standley KN, Takagi M, Maclean KH, et al. ATM-dependent suppression of stress signaling reduces vascular disease in metabolic syndrome. Cell Metab. 2006;4(5):377–89. doi: 10.1016/j.cmet.2006.10.002 17084711

[29] Miles PD, Treuner K, Latronica M, Olefsky JM, Barlow C. Impaired insulin secretion in a mouse model of ataxia telangiectasia. Am J Physiol Endocrinol Metab. 2007;293(1):E70–4. doi: 10.1152/ajpendo.00259.2006 17356010

[30] Connelly PJ, Smith N, Chadwick R, Exley AR, Shneerson JM, Pearson ER. Recessive mutations in the cancer gene Ataxia Telangiectasia Mutated (ATM), at a locus previously associated with metformin response, cause dysglycaemia and insulin resistance. Diabet Med. 2016;33(3):371–5. doi: 10.1111/dme.13037 26606753 PMC4832393

[31] Braun B, Eze P, Stephens BR, Hagobian TA, Sharoff CG, Chipkin SR, et al. Impact of metformin on peak aerobic capacity. Appl Physiol Nutr Metab. 2008;33(1):61–7. doi: 10.1139/H07-144 18347654

[32] Das S, Behera SK, Srinivasan A, Xavier AS, Selvarajan S, Kamalanathan S, et al. Effect of metformin on exercise capacity: A meta-analysis. Diabetes Res Clin Pract. 2018;144:270–8. doi: 10.1016/j.diabres.2018.08.022 30217594

[33] Malin SK, Stephens BR, Sharoff CG, Hagobian TA, Chipkin SR, Braun B. Metformin’s effect on exercise and postexercise substrate oxidation. Int J Sport Nutr Exerc Metab. 2010;20(1):63–71. doi: 10.1123/ijsnem.20.1.63 20190353

[34] Boule NG, Robert C, Bell GJ, Johnson ST, Bell RC, Lewanczuk RZ, et al. Metformin and exercise in type 2 diabetes: examining treatment modality interactions. Diabetes Care. 2011;34(7):1469–74. doi: 10.2337/dc10-2207 21602430 PMC3120188

[35] Painter RB, Young BR. Radiosensitivity in ataxia-telangiectasia: a new explanation. Proc Natl Acad Sci U S A. 1980;77(12):7315–7. doi: 10.1073/pnas.77.12.7315 6938978 PMC350493

