# Supplementary material for: The genetic association of the transcription factor NPAT with glycemic response to metformin involves regulation of fuel selection
Source: PLoS One. 2021 Jul 1;16(7):e0253533. doi: 10.1371/journal.pone.0253533 (PMC8248654; doi:10.1371/journal.pone.0253533)
Supplement: S1 Fig — (PDF) [file pone.0253533.s001.pdf]

Original blots for Fig1A. Samples of the lysates from mock-, empty vector- and NPAT plasmid-transfected HEK293 cells were run by SDS PAGE. HiMark pre-stained protein standards (LC5699) from Invitrogen were used. NPAT protein signals were detected using an ECL Western blotting analysis system and a Fuji medical X-ray film. The film was scanned on a CanoScan LiDE100 scanner and the image was saved as a JPG file.

NPAT ECL 10s
27/11/15
①+②

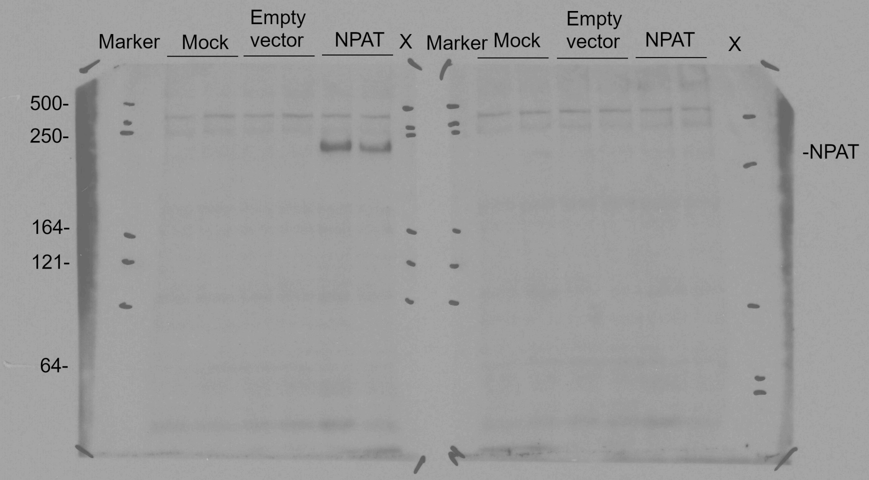

Original blot for Fig 1B left panel. HEK293 cells were transfected with NPAT plasmids for 48 hr. Cells were lysed in Urea lysis buffer. Samples of the lysates were run by SDS PAGE. HiMark pre-stained protein standards (LC5699) from Invitrogen were used. NPAT protein signals were detected using an ECL Western blotting analysis system and a Fuji medical X-ray film. The film was scanned on a CanoScan LiDE100 scanner and the image was saved a JPG file.

NPAT ECL 10s
27/11/15
(1) + (2)

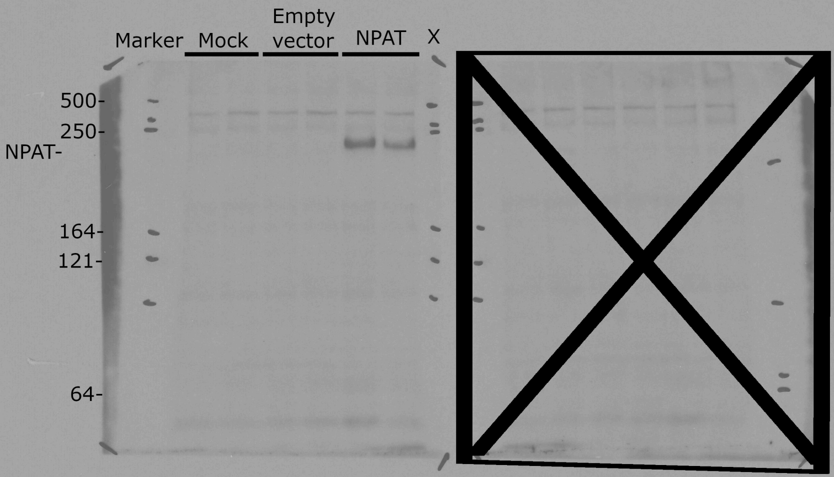

NPAT ECL 3s
27/11/15
2a+2b

Original blot for Fig 1B middle panel. HEK293 cells were transfected with NPAT plasmids for 48 hr. Cells were lysed in Urea lysis buffer. Samples of the lysates were run by SDS-PAGE and proteins were transferred on a PVDF membrane. HiMark pre-stained protein standards were used. NPAT protein signals were detected using an ECL Western blotting analysis system and a Fuji medical film. The film was sacnned on a CanoScan LiDE100 scanner and the image was saved as a JPG file.

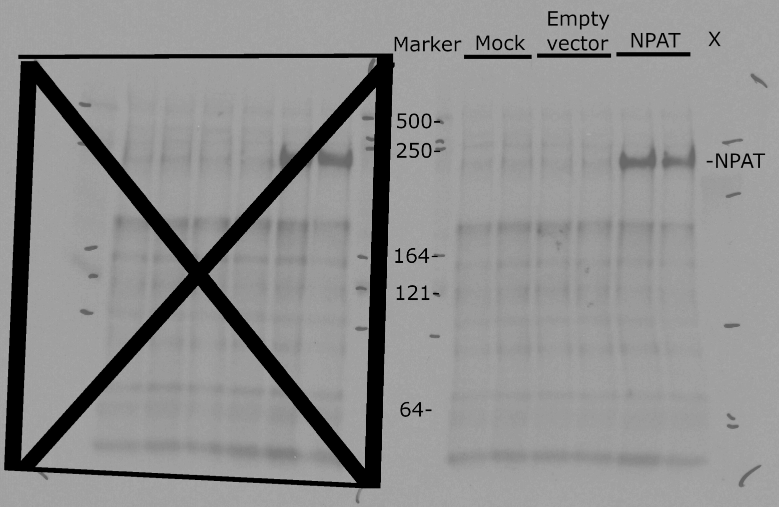

Original blot for Fig 1B right panel. HEK293 cells were transfected with NPAT plasmids for 48 hr. Cells were lysed in Urea lysis buffer. Samples of the lysates were run by SDS-PAGE and proteins were transferred on a PVDF membrane. HiMark pre-stained protein standards (LC5699) from Invitrogen were used. NPAT protein signals were detected using an ECL Western blotting analysis system and a Fuji medical X-ray film. The film was scanned on a CanoScan LiDE100 scanner and the image was saved as a JPG file.

NPAT ECL prime 15s 05/11/15

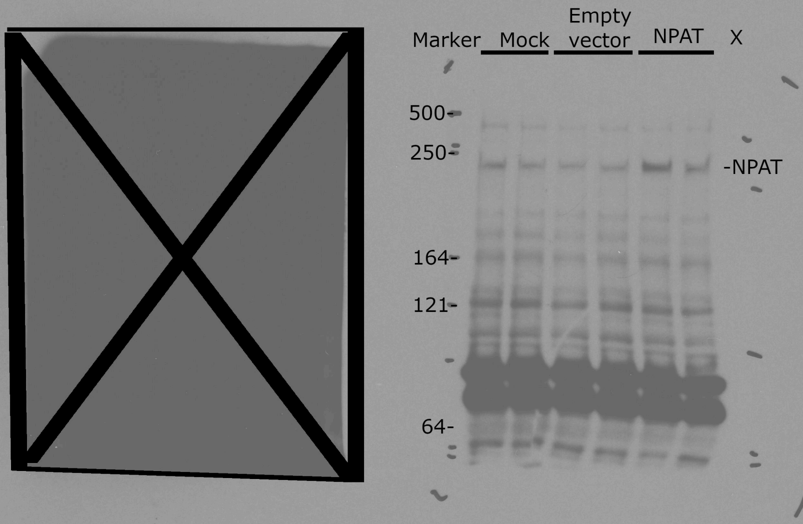

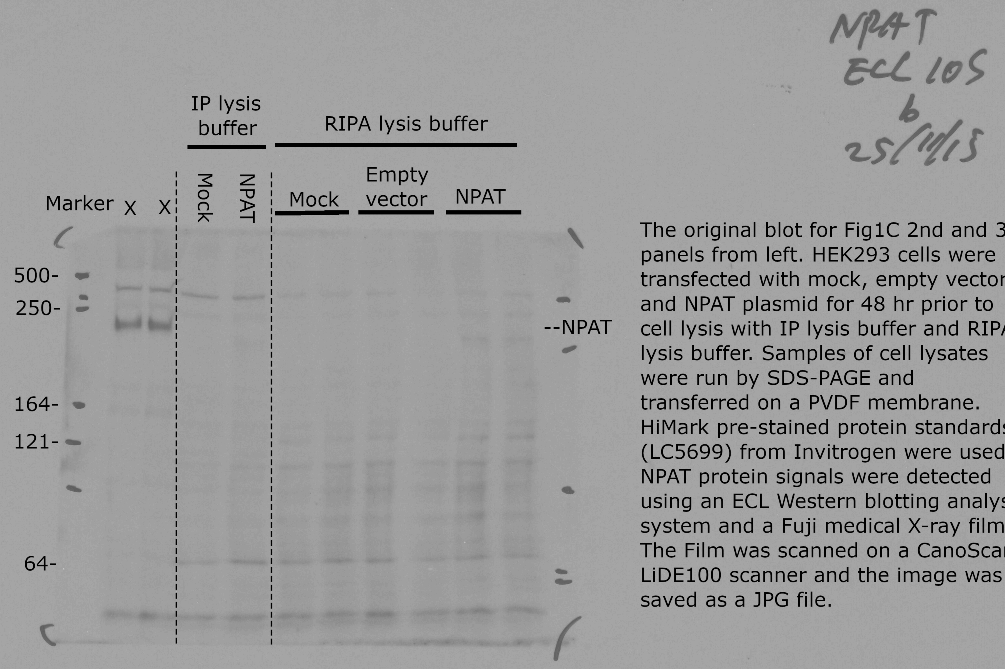

The original blot for Fig1C 2nd and 3rd panels from left. HEK293 cells were transfected with mock, empty vector and NPAT plasmid for 48 hr prior to cell lysis with IP lysis buffer and RIPA lysis buffer. Samples of cell lysates were run by SDS-PAGE and transferred on a PVDF membrane. HiMark pre-stained protein standards (LC5699) from Invitrogen were used. NPAT protein signals were detected using an ECL Western blotting analysis system and a Fuji medical X-ray film. The Film was scanned on a CanoScan LiDE100 scanner and the image was saved as a JPG file.

Original blots for Fig1C left and right panels. HEK293 cells were transfected with NPAT plasmids for 48 hr prior to cell lysis with Tris-Triton lysis buffer and Urea lysis buffer. Samples of the lysates were run by SDS-PAGE and proteins were transferred on PVDF membranes. HiMark pre-stained protein standards (LC5699) from Invitrogen were used. NPAT protein signals were detected using an ECL Western blotting analysis system and a Fuji medical X-ray film. The film was scanned on a CanoScan LiDE100 scanner and the image was saved as a JPG file.

NPAT
ECL 20s
25/11/15
a+C

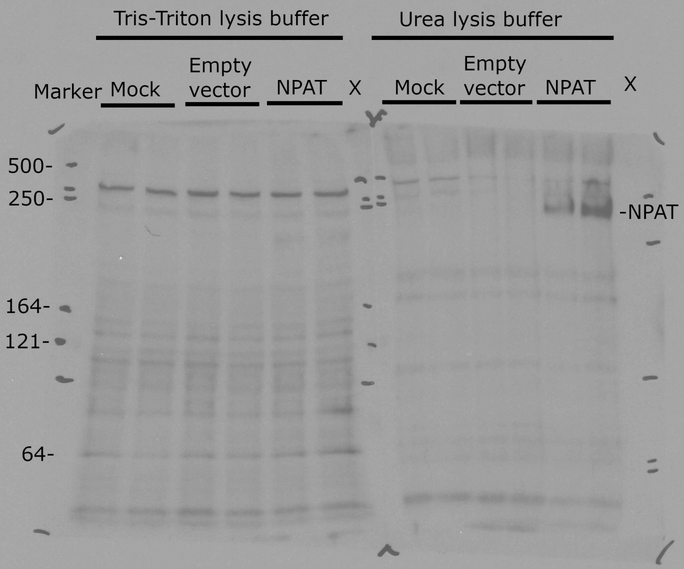

Original blots for Fig1D. HEK293 cells were transfected with mock, empty vector and NPAT plasmid for 48 hr prior to cell lysis with Tris-Triton lysis buffer, IP lysis buffer and RIPA lysis buffer. The resulting insoluble pellets were further lysed in Urea lysis buffer. Samples of these lysates were run by SDS-PAGE and proteins were transferred on PVDF membranes. HiMark pre-stained protein standards (LC5699) from Invitrogen were used.

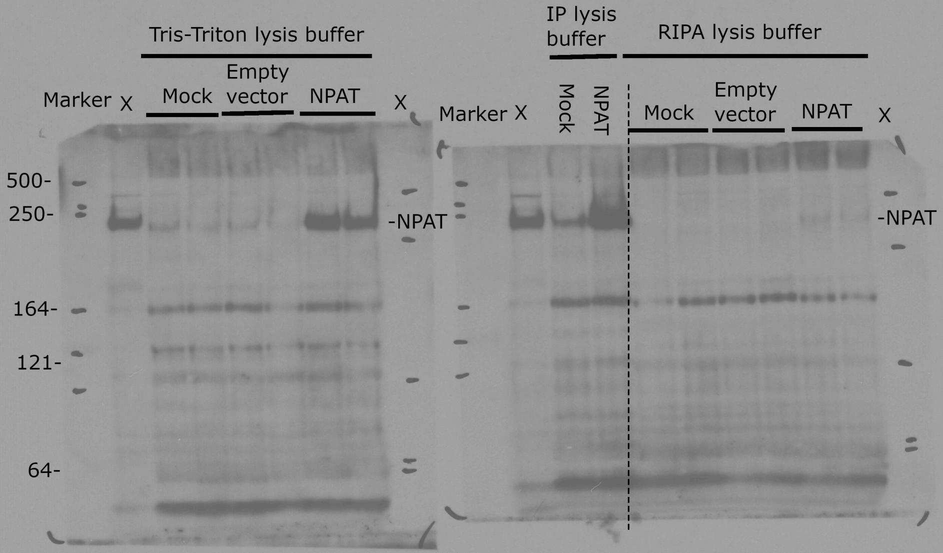

NPAT protein signals were detected using ECL Western blotting analysis system and Fuji medical X-ray films. Films were scanned on a CanoScan LiDE100 scanner and images were saved as JPG files.

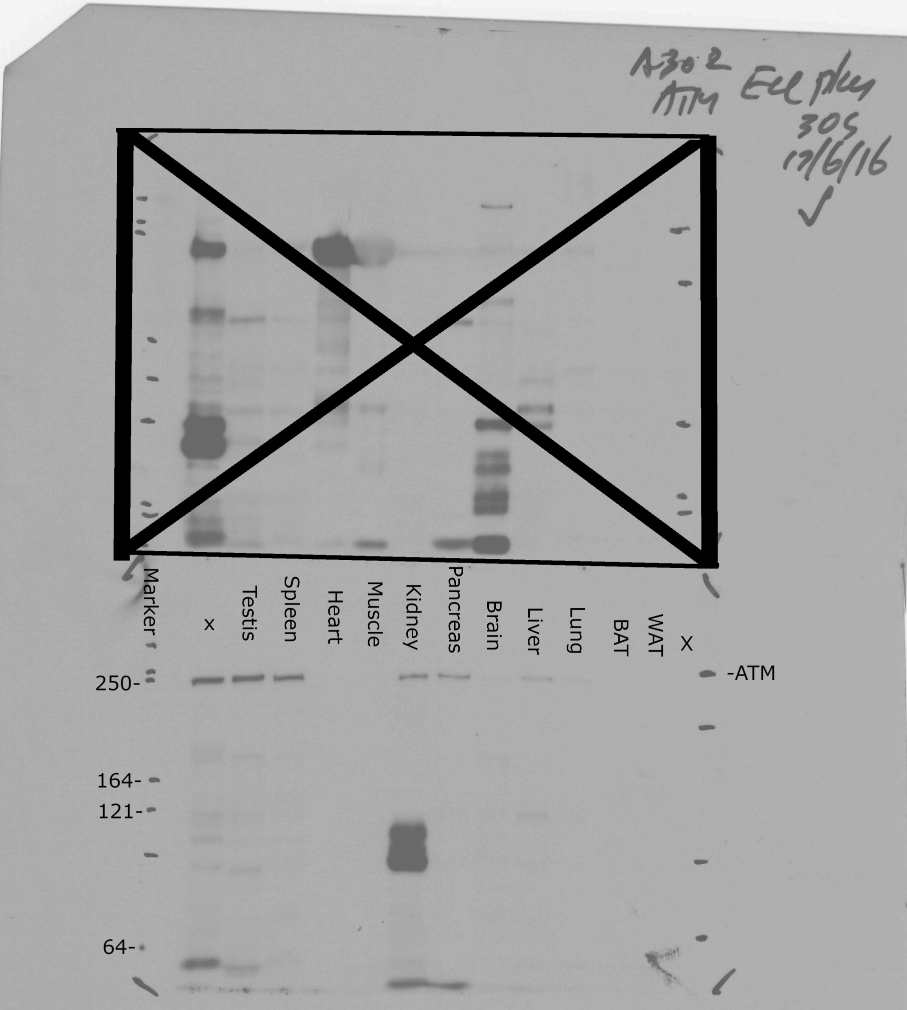

Original blot for Fig2C ATM. Mouse tissues were lysed in Urea lysis buffer and samples of the lysates were run by SDS-PAGE. Proteins were transferred on a PVDF membrane. HiMark pre-stained protein standards (LC5699) from Invitrogen were used. ATM protein signals were detected using an ECL Western blotting analysis system and a Fuji medical X-ray film. The film was scanned on a CanoScan LiDE100 scanner and the image was saved as a JPG file.

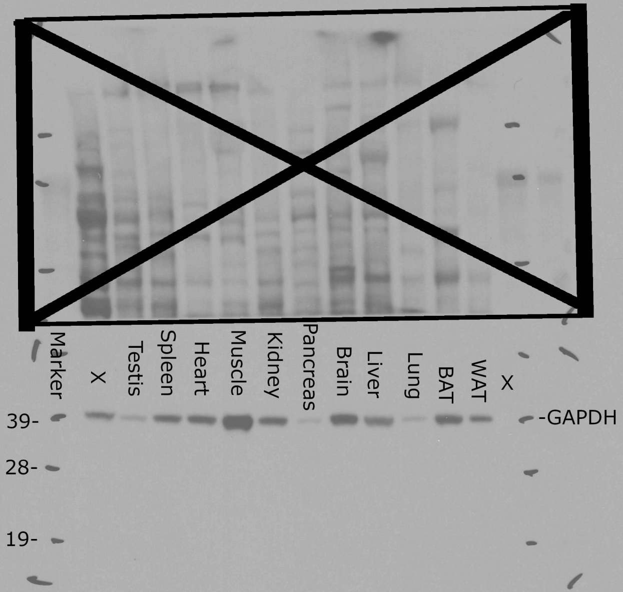

Original blot for Fig 2C GAPDH. Mouse tissues were lysed in Urea lysis buffer and samples of the lysates were run on a Novex NuPAGE 4-12% Bis-Tris gel. Proteins were transferred on a PVDF membrane. SeebluePlus2 pre-stained protein standards (LC5925) from Invitrogen were used. Protein signals were detected using an ECL Western blotting analysis system and a Fuji medical X-ray film. The film was scanned on a CanoScan LiDE100 scanner and the image was saved as a JPG file.

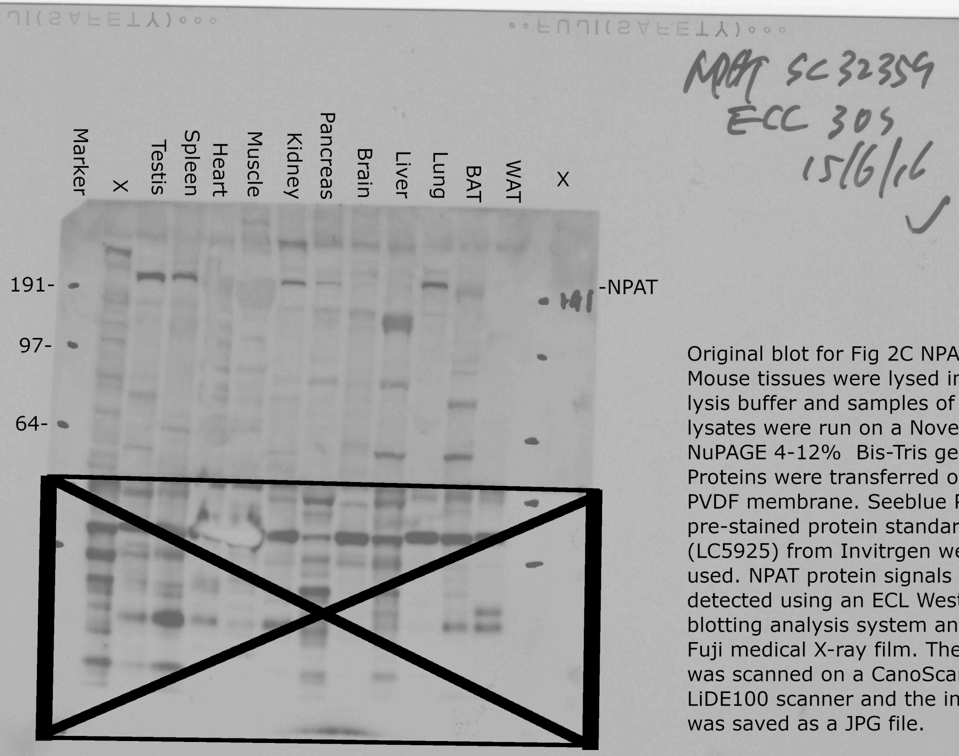

Original blot for Fig 2C NPAT. Mouse tissues were lysed in Urea lysis buffer and samples of the lysates were run on a Novex NuPAGE 4-12% Bis-Tris gel. Proteins were transferred on a PVDF membrane. Seeblue Plus2 pre-stained protein standards (LC5925) from Invitrgen were used. NPAT protein signals were detected using an ECL Western blotting analysis system and a Fuji medical X-ray film. The film was scanned on a CanoScan LiDE100 scanner and the image was saved as a JPG file.

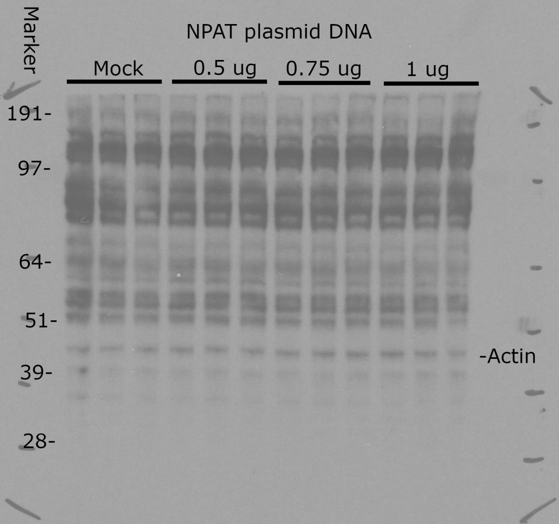

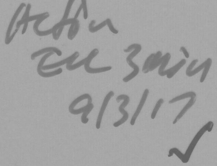

Original blot for Fig 3Ai actin. HEK293 cells were transfected with 0.5 ug, 0.75 ug and 1 ug of NPAT plasmid DNA for 24 hr. Cells were lysed in Urea lysis buffer. Samples of the lysates were run on a Novex NuPAGE 4-12% Bis-Tris gel and proteins were transferred on a PVDF membrane. SeebluePlus2 pre-stained protein standards (LC5925) from Invitrogen were used. Actin protein signals were detected using an ECL Western blotting analysis system and a Fuji medical X-ray film. The film was scanned on a CanoScan LiDE100 scanner and the image was saved as a JPG file.

ATM
ECL prime
9/3/17

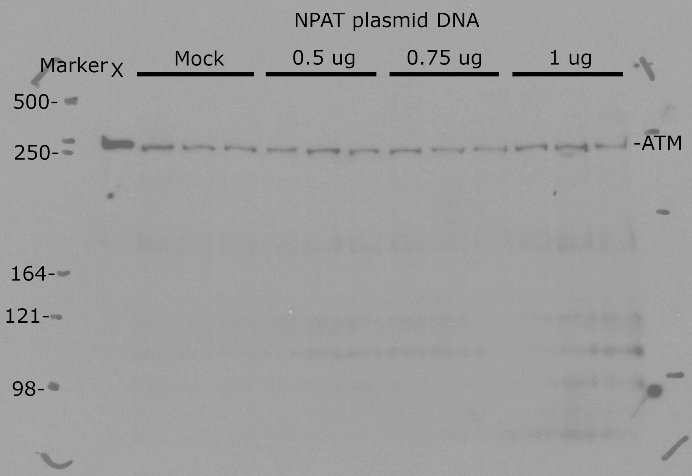

Original blot for Fig 3Ai ATM. HEK293 cells were transfected with 0.5 ug, 0.75 ug and 1 ug of NPAT plasmid DNA for 24 hr. Cells were lysed in Urea lysis buffer. Samples of the lysates were run by SDS-PAGE and proteins were transferred on a PVDF membrane. HiMark pre-stained protein standards (LC5699) from Invitrogen were used. ATM protein signals were detected using an ECL Western blotting analysis system and a Fuji medical X-ray film. The film was scanned on a CanoScan LiDE100 scanner and the image was saved as a JPG file.

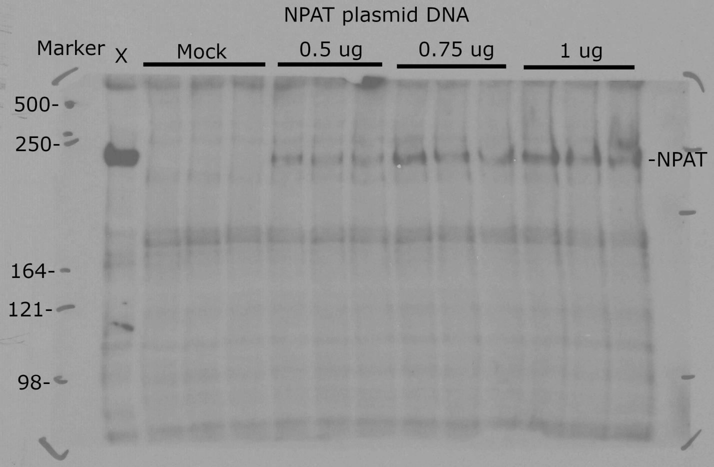

Original blot for Fig 3Ai NPAT. HEK293 cells were transfected with 0.5 ug, 0.75 ug and 1 ug of NPAT plasmid DNA for 24 hr. Cells were lysed in Urea lysis buffer. Samples of the lysates were run by SDS-PAGE. Proteins were transferred on a PVDF membrane. HiMarker pre-stained protein standards (LC5699) from Invitrogen were used. NPAT protein signals were detected using an ECL Western blotting analysis system and a Fuji medical X-ray film. The film was scanned on a CanoScan LiDE100 scanner and the image was saved as a JPG file.

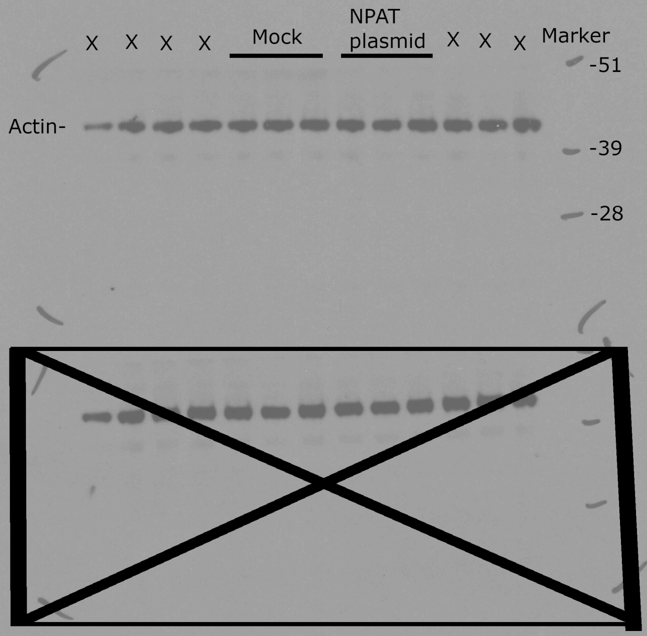

Original blot for Fig 3Bi actin. HEK293 cells were transfected with NPAT plasmid DNA for 24 hr. Cells were lysed in Urea lysis buffer. Samples of the lysates were run on a Novex NuPAGE 4-12% Bis-Tris gel and proteins were transferred on a PVDF membrane. SeebluePlus2 pre-stained protein standards (LC5925) from Invitrogen were used. Actin protein signals were detected usin an ECL Western blotting analysis system and a Fuji medical X-ray film. The film was scanned on a CanoScan LiDE100 scanner and the image was saved as a JPG file.

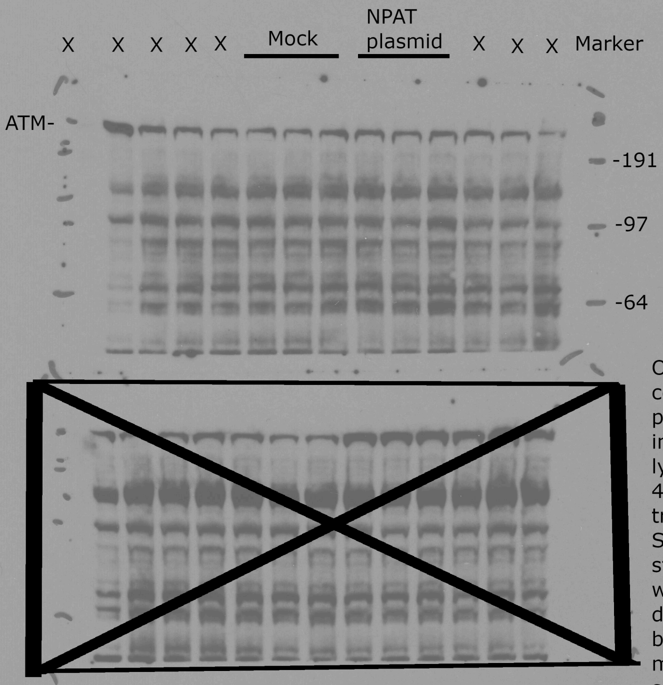

Original blot for Fig3Bi ATM. HEK293 cells were transfected with NPAT plasmid DNA for 24hr. Cells were lysed in Urea lysis buffer. Samples of the lysates were run on a Novex NuPAGE 4-12% Bis-Tris gel and proteins were transferred on a PVDF membrane. SeebluePlus2 pre-stained protein standards (LC5925) from Invitrogen were used. ATM protein signals were detected using an ECL Western blotting analysis system and a Fuji medical X-ray film. The film was scanned on a CanoScan LiDE100 scanner and the image was saved as a JPG file

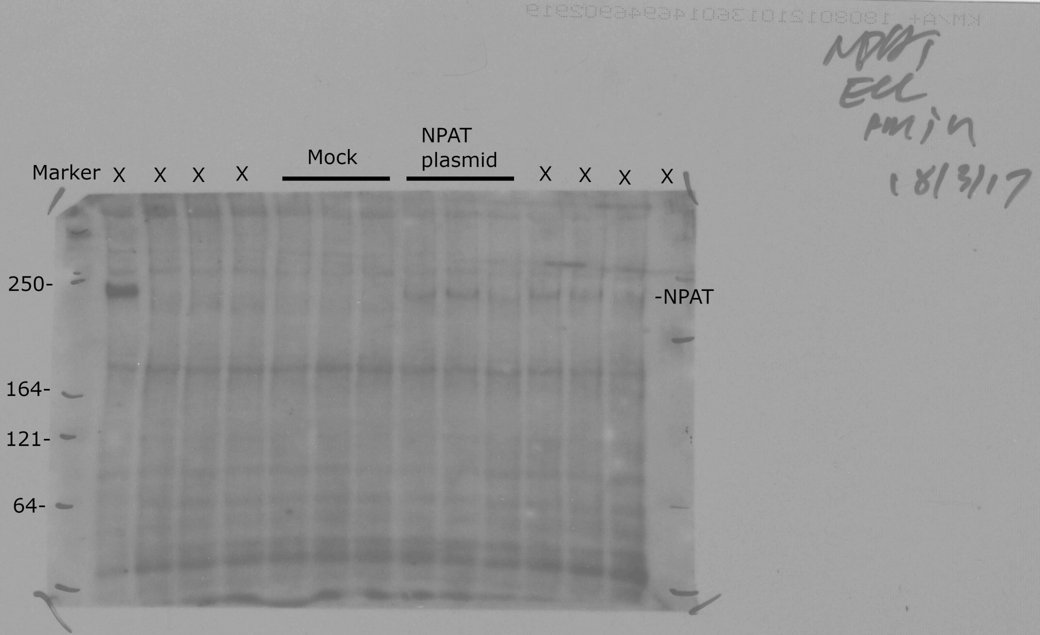

Original blot for Fig 3Bi NPAT. HEK293 cells were transfected with NPAT plasmid DNA for 24 hr and cells were lysed in Urea lysis buffer. Samples of the lysates were run by SDS-PAGE and the proteins were transferred on a PVDF membrane. HiMark pre-stained protein standards (LC5699) from Invitrogen were used. NPAT protein signals were detected using an ECL Western blotting system and a Fuji medical X-ray film. The film was scanned on a CanoScan LiDE100 scanner and the image was saved as a JPG file

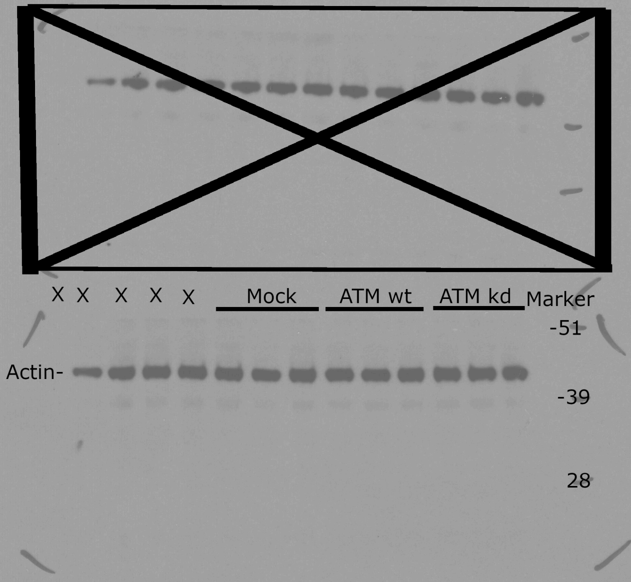

Original blot for Fig 3Ci actin. HEK293 cells were transfected with plasmids encoding ATM wild-type (ATM wt) and ATM kinase dead mutation (ATM kd) for 24 hr. Cells were lysed in Urea lysis buffer. Samples of the lysates were run on a Novex NuPAG 4-12% Bis-Tris gel and proteins were transferred on a PVDF membrane. SeeBlue Plus2 pre-stained protein standards (LC5925) from Invitrogen were used. Actin protein signals were detected using an ECL Western blotting analysis system and a Fuji medical X-ray film. The film was scanned on a CanoScan LiDE100 scanner and the image was saved as a JPG file.

Gel 1a+2a
[illegible]
ATM
20/4/17
2a

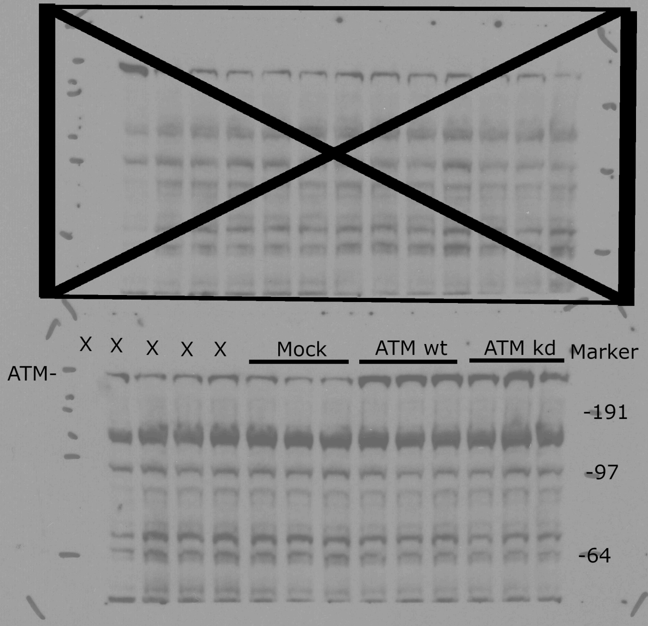

Original blot for Fig 3Ci ATM. HEK293 cells were transfcted with plasmids encoding ATM wild-type (ATM wt) or ATM with kinase dead mutation (ATM kd) for 24 hr. Cells were lysed in Urea lysis buffer. Samples of the lysates were run on a Novex NuPAGE 4-12% Bis-Tris gel and proteins were transferred on a PVDF membrane. SeeBlue Plus 2 pre-stained protein standards (LC5925) from Invitrogen were used. ATM protein signals were detected using an ECL Western blotting analysis system and a Fuji medical X-ray film. The film was scanned on a CanoScan LiDE100 scanner and the image was saved as a JPG file.

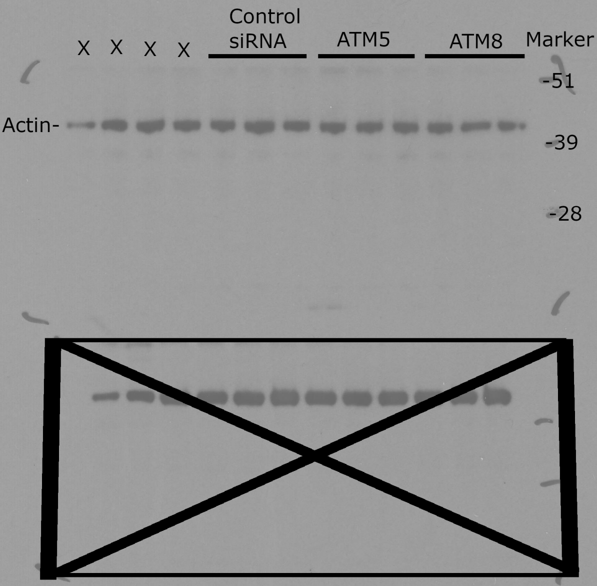

Original blot for Fig 3Di actin. HEK293 cells were transfected with ATM siRNA, ATM5 or ATM8 for 48 hr. Cells were lysed in Urea lysis buffer. Samples of the lysates were run on a Novex NuPAGE 4-12% Bis-Tris gel and proteins were transferred on a PVDF membrane. SeeBlue Plus2 pre-stained protein standards (LC5925) from Invitrogen were used. Actin protein signals were detected using an ECL Western blotting analysis system and a Fuji medical X-ray film. The film was scanned on a CanoScan LiDE100 scanner and the image was saved as a JPG file.

[illegible]
[illegible]
15/4/17

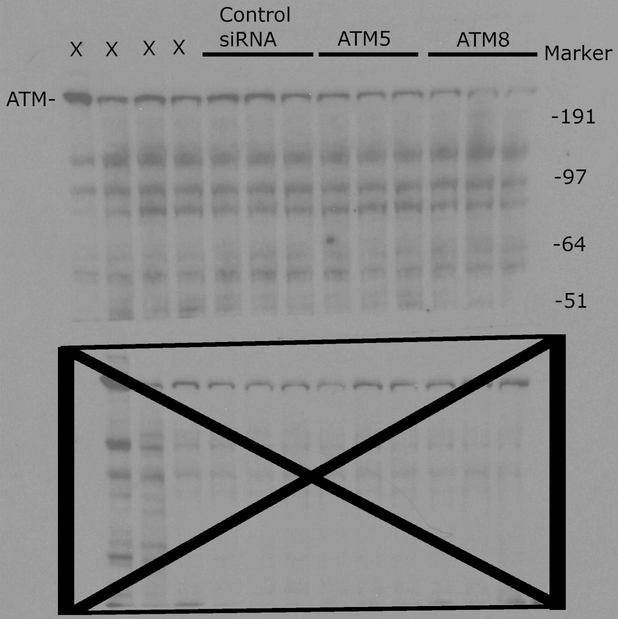

Original blot for Fig 3Di ATM. HEK293 cells were transfected with ATM siRNA, ATM5 and ATM8 for 48 hr. Cells were lysed in Urea lysis buffer. Samples of the lysates were run on a Novex NuPAGE 4-12% gel and proteins were transferred on a PVDF membrane. SeeBlue Plus2 pre-stained protein standards (LC5925) from Invitrogen were used. ATM protein signals were detected using an ECL Western blotting analysis system and a Fuji medical X-ray film. The film was scanned on a CanoScan LiDE100 scanner and the image was saved as a JPG file.

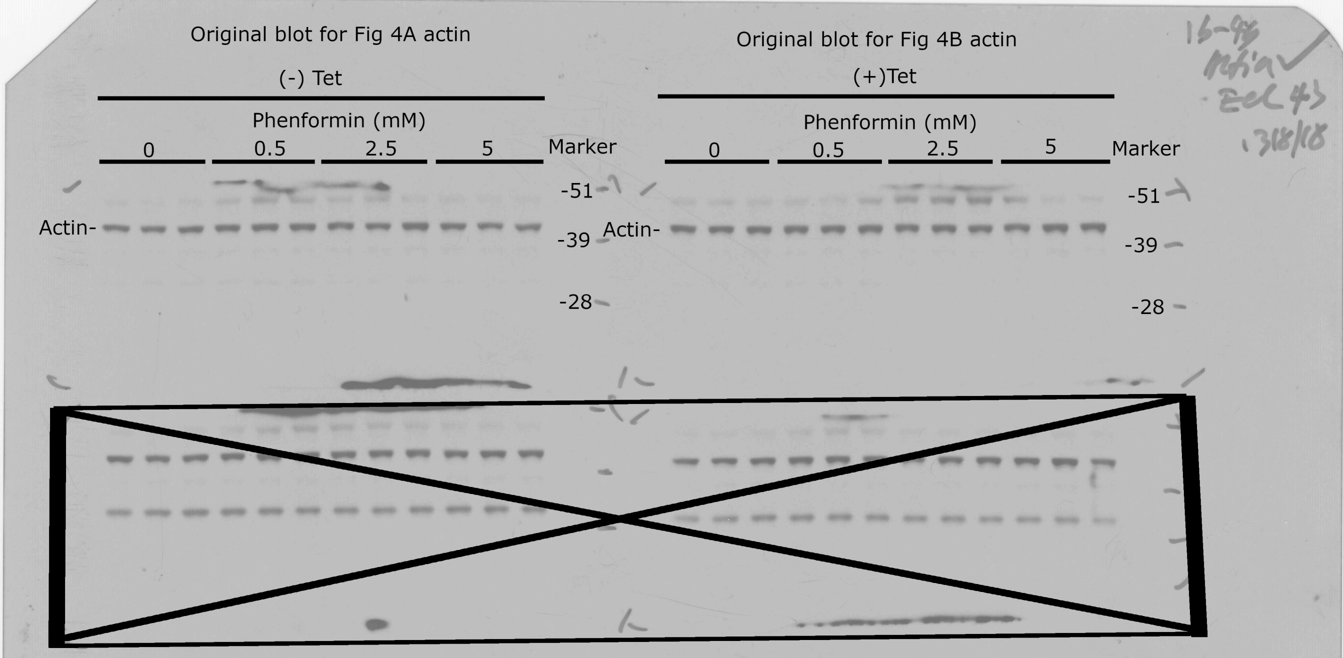

Original blots for Fig 4A&B actin. Stable HEK293 cells were treated with phenformin in the absence (-Tet) or presence (+Tet) of tetracycline (0.1 ug/ml) for 6 hr. Cells were lysed in Tris-Triton lysis buffer. Samples of the lysates were run on Novex NuPAGE 4-12% Bis-Tris gels and proteins were transferred on PVDF membranes. SeeBluePlus2 pre-stained protein standards (LC5925) from Invitrogen were used. Actin protein signals were detected using an ECL Western blotting analysis system and a Fuji medical X-ray film. The film was scanned on a CanoScan LiDE100 scanner and the image was saved as a JPG file.

Original blots for Fig 4A&B AMPKa. Stable HEK293 cells were treated with phenformin in the absence (-Tet) or presence (+Tet) of tetracycline (0.1 ug/ml) for 6 hr. Cells were lysed in Tris-Triton lysis buffer. Samples of the lysates were run on Novex NuPAGE 4-12% Bis-Tris gels and proteins were transferred on PVDF membranes. SeeBluePlus2 pre-stained protein standards (LC5925) from Invitrogen were used. AMPKa protein signals were detected using an ECL Western blotting analysis system and a Fuji medical X-ray film. The film was scanned on a CanoScan LiDE100 scanner and the image was saved as a JPG file.

1a-4a
AMPKa
ECL 2min
11/8/18

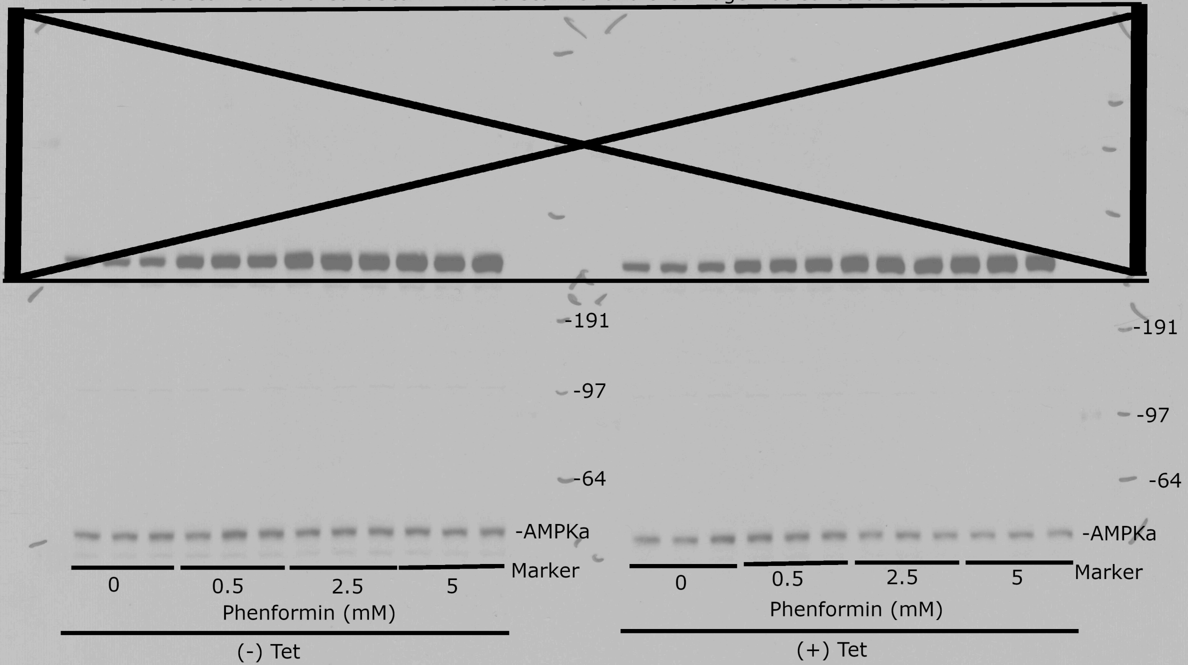

Original blot for Fig 4A AMPKa

Original blot for Fig 4B AMPKa

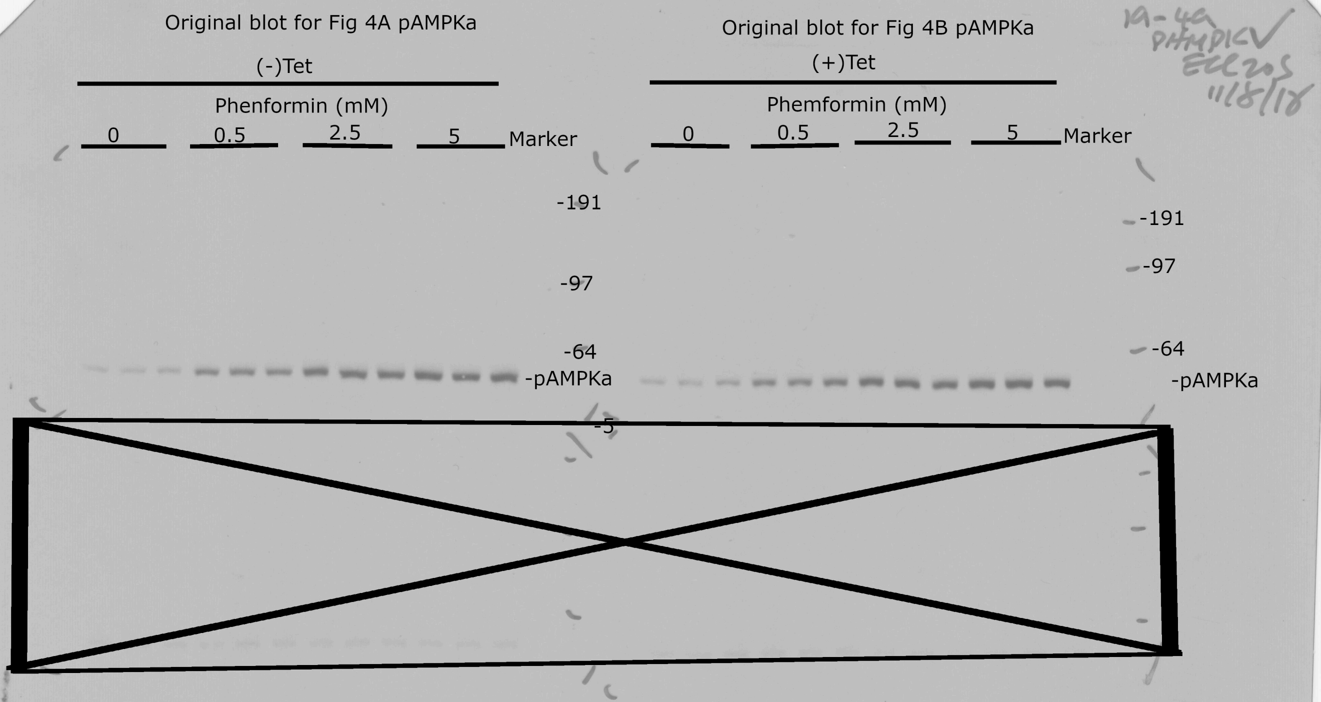

Original blots for Fig 4A&B pAMPKa. Stable HEK293 cells were treated with phenformin in the absence (-Tet) or presence (+Tet) of tetracycline (0.1ug/ml) for 6 hr. Cells were lysed in Tris-Triton lysis buffer. Samples of the lysates were run on Novex NuPAGE 4-12% Bis-Tris gels and proteins were transferred on PVDF membranes. SeeBluePlus2 pre-stained protein standards (LC5925) from Invitrogen were used. pAMPKa protein signals were detected using an ECL Western blotting analysis system and a Fuji medical X-ray film. The film was scanned on a CanoScan LiDE100 scanner and the image was saved as a JPG file.

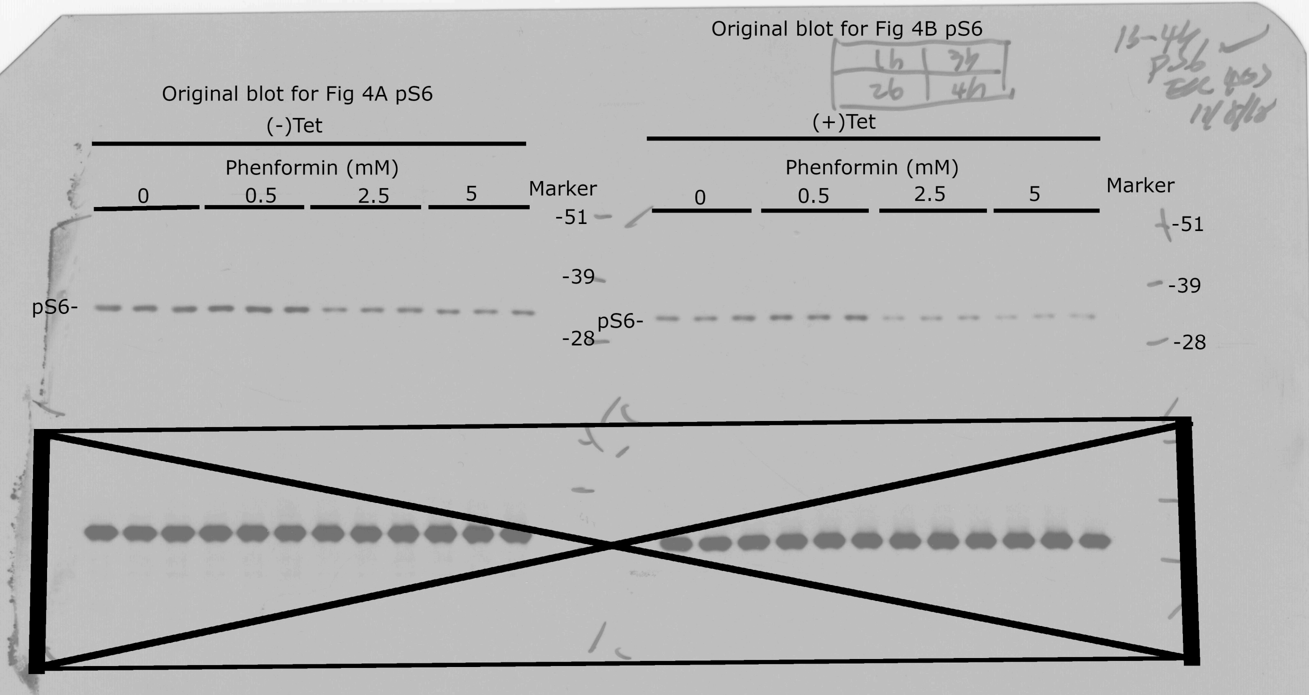

Original blots for Fig 4A&B pS6. Stable HEK293 cells were treated with phenformin in the absence (-Tet) or the presence (+Tet) of tetracycline (0.1 ug/ml) for 6 hr. Cells were lysed in Tris-Triton lysis buffer. Samples of the lysates were run on Novex NuPAGE 4-12% Bis-Tris gels and proteins were transferred on PVDF membranes. SeeBlue pre-stained protein standards (LC5925) from Invitrogen were used. pS6 protein signals were detected using an ECL Western blotting analysis system and a Fuji medical X-ray film. The film was scanned on a CanoScan LiDE100 scanner and the image was saved as a JPG file.

Original blots for Fig 4A&B S6. Stable HEK293 cells were treated with phenformin in the absence (-Tet) or presence (+Tet) of tetracycline (0.1 ug/ml) for 6 hr. Cells were lysed in Tris-Triton lysis buffer. Samples of the lysates were run on Novex NuPAGE 4-12% Bis-Tris gels and proteins were transferred on PVDF membranes. SeeBluePlus2 pre-stained protein standards (LC5925) from Invitrogen were used. S6 protein signals were detected using an ECL Western blotting analysis system and a Fuji medical X-ray film. The film was scanned on a Canoscan LiDE100 scanner and the image was saved as a JPG file.

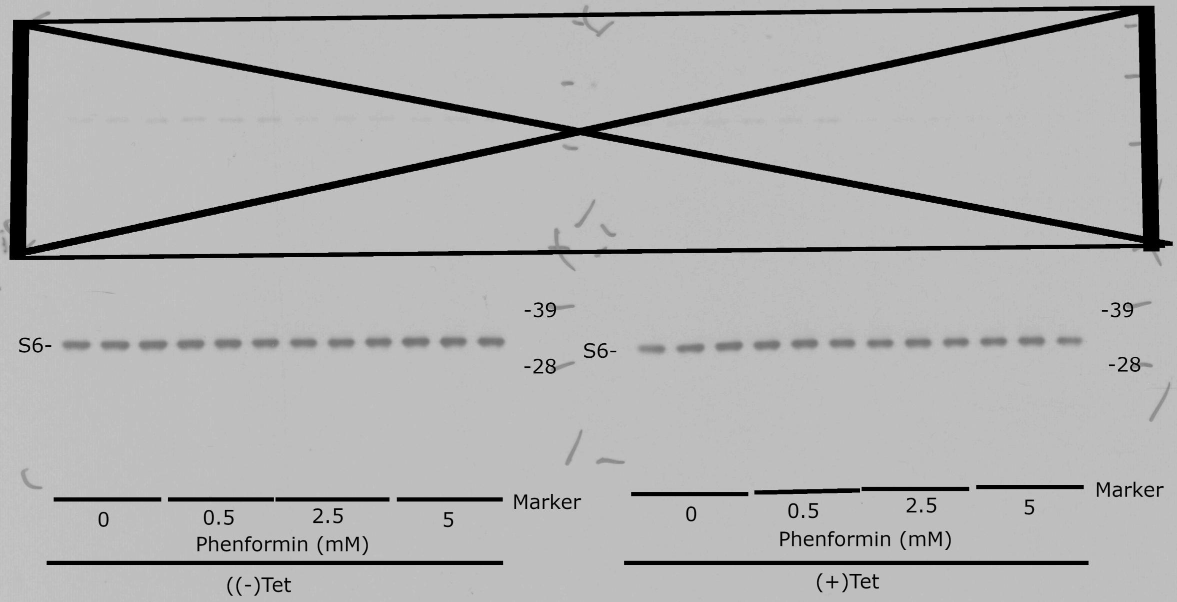

Original blot for Fig 4A S6

Original blot for Fig 4B S6.

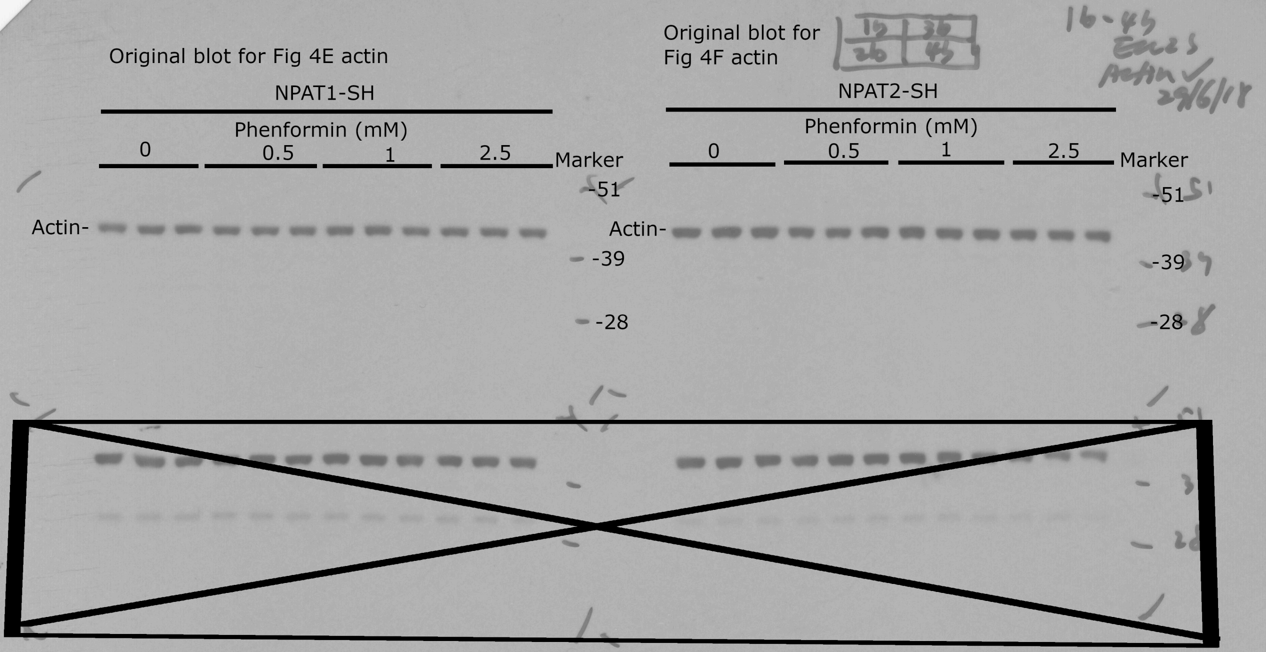

Original blots for Fig 4E&F actin. Stable NPAT1-SH and NPAT2-SH cells were treated with phenformin for 6 hr. Cells were lysed in Tris-Triton lysis buffer. Samples of the lysates were run on Novex NuPAGE 4-12% Bis-Tris gels and proteins were transferred on PVDF membranes. SeeBluePlus2 pre-stained protein standards (LC5925) from Invitrogen were used. Actin protein signals were detected using an ECL Western blotting analysis system and a Fuji medical X-ray film. The film was scanned on a CanoScan LiDE100 scanner and the image was saved as a JPG file.

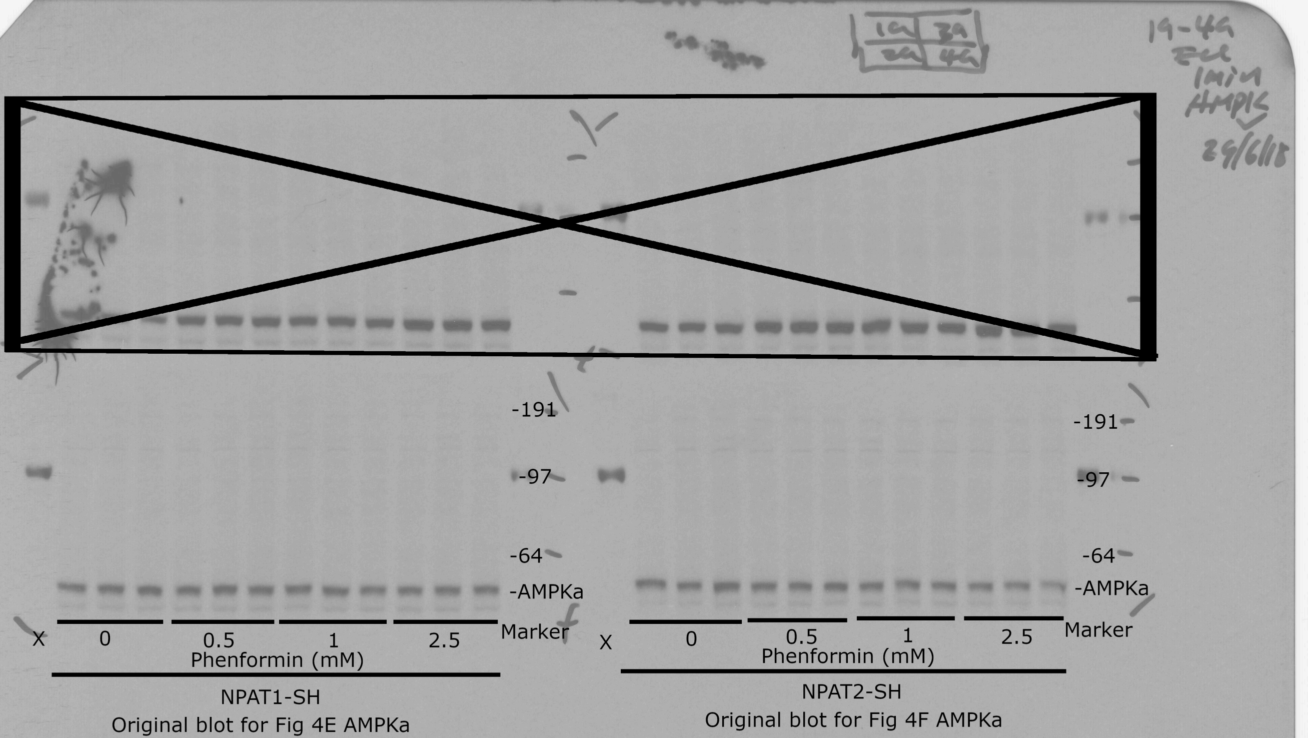

Original blots for Fig 4E&F AMPKa. Stable NPAT1-SH and NPAT2-SH cells were treated with phenformin for 6 hr. Cells were lysed in Tris-Triton lysis buffer. Samples of the lysates were run on Novex NuPAGE 4-12% Bis-Tris gels and proteins were transferred on PVDF membranes. SeeBlue pre-stained protein standards (LC5925) from Invitrogen were used. AMPKa protein signals were detected using an ECL Western blotting analysis system and a Fuji medical X-ray film. The film wasd scanned on a CanoScan LiDE100 scanner and the image was saved as a JPG file.

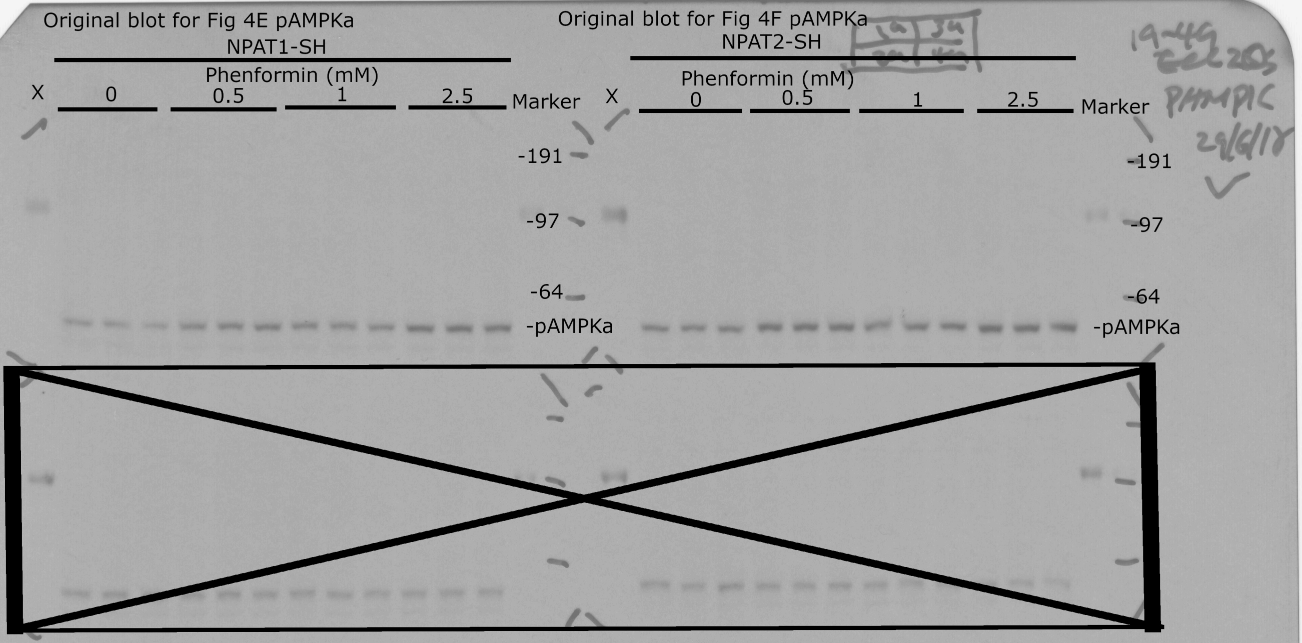

Original blots for Fig 4E&F pAMPKa. Stable NPAT1-SH and NPAT2-SH cells were treated with phenformin for 6 hr. Cells were lysed in Tris-Triton lysis buffer. Samples of the lysates were run on Novex NuPAGE 4-12% Bis-Tris gels and proteins were transferred on PVDF membranes. SeeBluePlus2 pre-stained protein standards (LC5925) from Invitrogen were used. pAMPKa protein signals were detected using an ECL Western blotting analysis system and a Fuji medical X-ray film. The film was scanned on a CanoScan LiDE100 scanner and the image was saved as a JPG file.

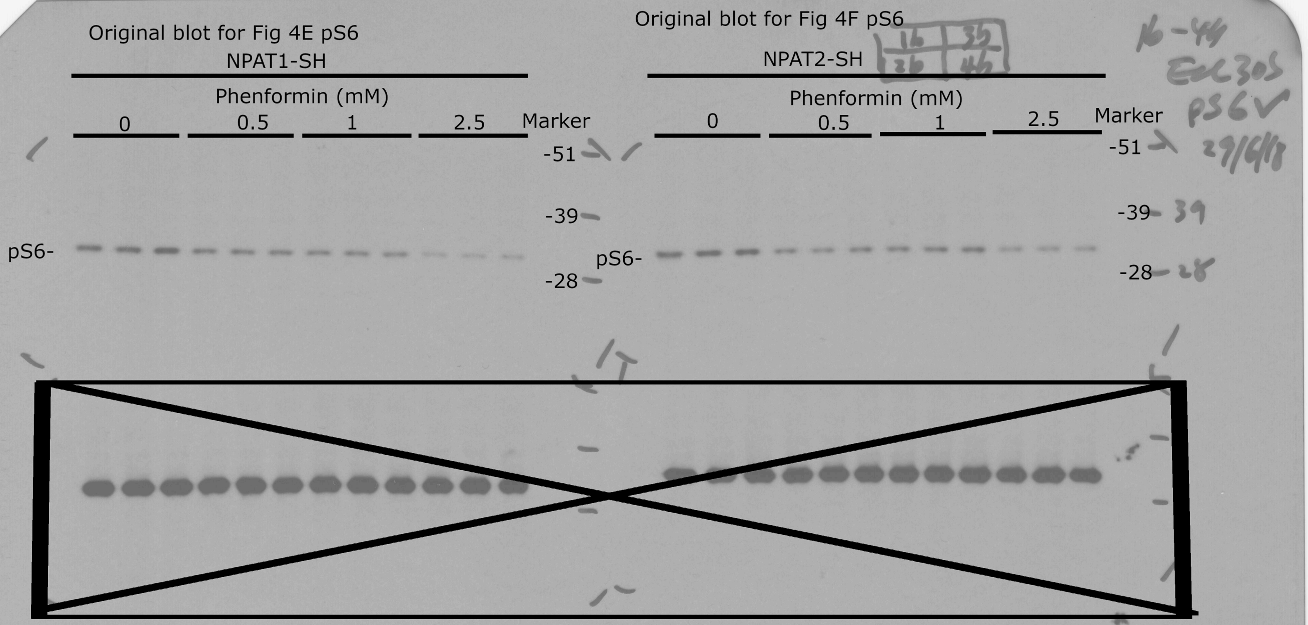

Original blots for Fig 4E&F pS6. Stable NPAT1-SH and NPAT2-SH cells were treated with phenformin for 6 hr. Cells were lysed in Tris-Triton lysis buffer. Samples of the lysates were run on Novex NuPAGE 4-12% Bis-Tris gels and proteins were transferred on PVDF membranes. SeeBluePlus2 pre-stained protein standards (LC5925) from Invitrogen were used. pS6 protein signals were detected using an ECL Western blotting analysis system and a Fuji medical X-ray film. The film was scanned on a CanoScan LiDE100 scanner and the image was saved as a JPG file.

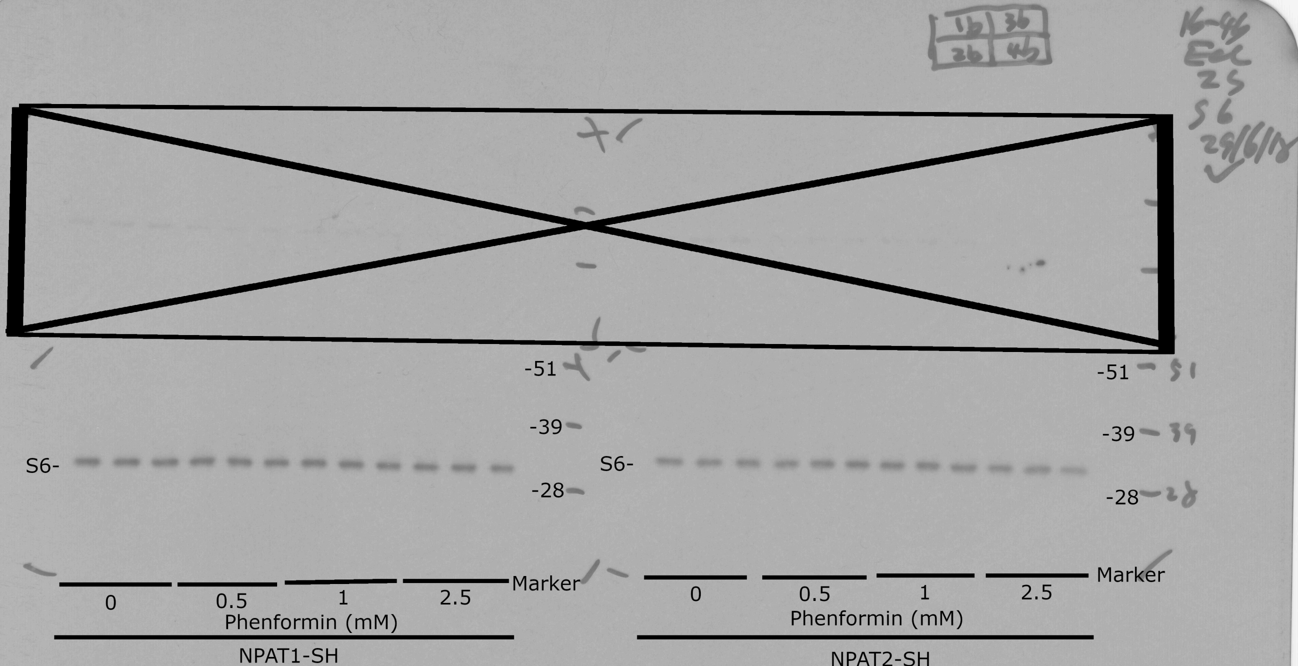

Original blot for Fig 4E S6

Original blot for Fig 4F S6

Original blots for Fig 4E&F S6. Stable NPAT1-SH and NPAT2-SH were treated with phenformin for 6 hr. Cells were lysed in Tris-Triton lysis buffer. Samples of the lysates were run on Novex NuPAGE 4-12% Bis-Tris gels and proteins were transferred on PVDF membranes. SeeBluePlus2 pre-stained protein standards (LC5925) from Invitrogen were used. S6 protein signals were detected using an ECL Western blotting analysis system and a Fuji medical X-ray film. The film was scanned on a CanoScan LiDE100 scanner and the image was saved as a JPG file.

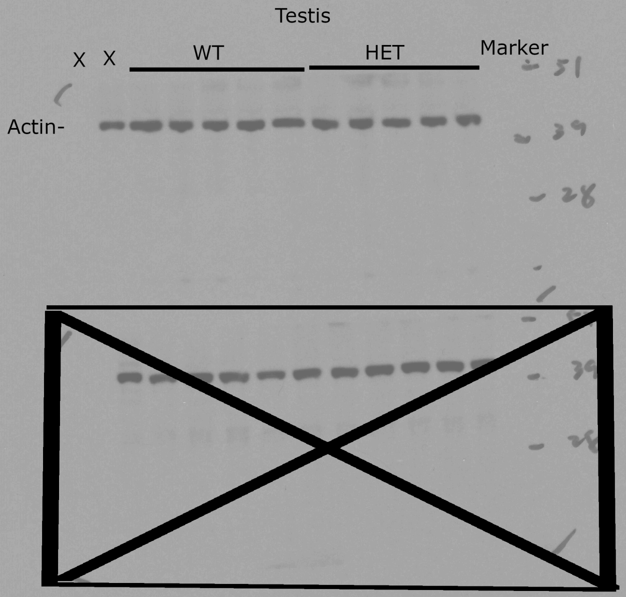

Original blot for Fig 5C actin. Testis tissues from wild-type (WT) and heterozygous (HET) NPAT(+/-) mice were lysed in Urea lysis buffer. Samples of the lysates were run on a Novex NuPAGE 4-12% Bis-Tris gel and proteins were transferred on a PVDF membrane. SeeBluePlus2 pre-stained protein standards (LC5925) from Invitrogen were used. Actin protein signals were detected using an ECL Western blotting analysis system and a Fuji medical X-ray film. The film was scanned on a CanoScan LiDE100 scanner and the image was saved as a JPG file.

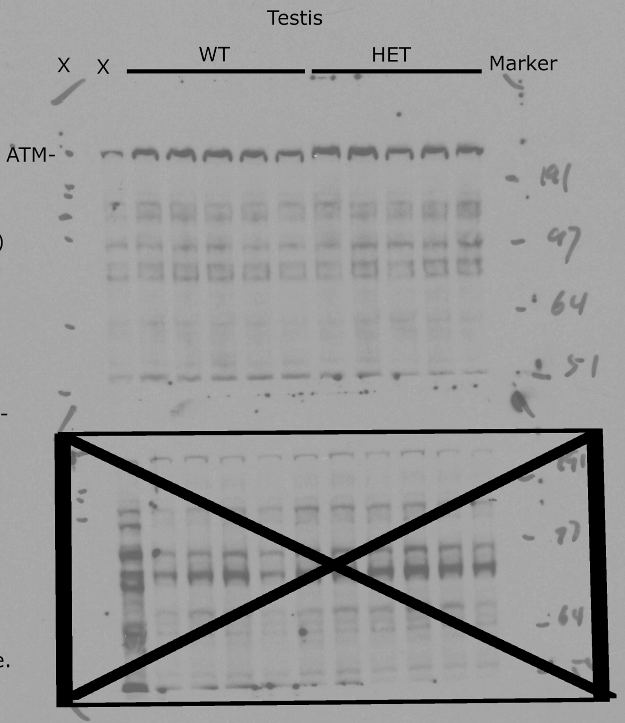

Original blot for Fig 5C ATM. Testis tissues from wild-type (WT) and heterozygous (HET) NPAT(+/-) mice were lysed in Urea lysis buffer. Samples of the lysates were run on a Novex NuPAGE 4-12% Bis-Tris gel and proteins were transferred on a PVDF membrane. SeeBluePlus2 pre-stained protein standards (LC5925) from Invitrogen were used. ATM protein signals were detected using an ECL Western blotting analysis system and a Fuji medical X-ray film. The film was scanned on a CanoScan LiDE100 scanner and the image was saved as a JPG file.

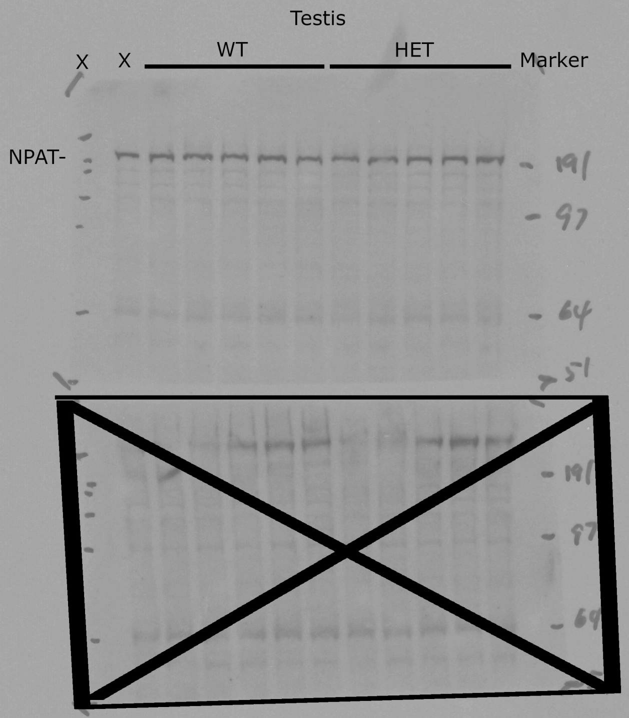

Gel 1a+2a
4/3/17

Original blot for Fig 5C NPAT. Testis tissues from wild-type (WT) and heterozygous (HET) NPAT(+/-) mice were lysed in Urea lysis buffer. Samples of the lysates were run on a Novex NuPAGE 4-12% Bis-Tris gel and proteins were transferred on a PVDF membrane. SeeBlue pre-stained protein standards (LC5925) from Invitrogen were used. NPAT protein signals were detected using an ECL Western blotting analysis system and a Fuji medical X-ray film. The film was scanned on a CanoScan LiDE100 scanner and the image was saved as a JPG file.

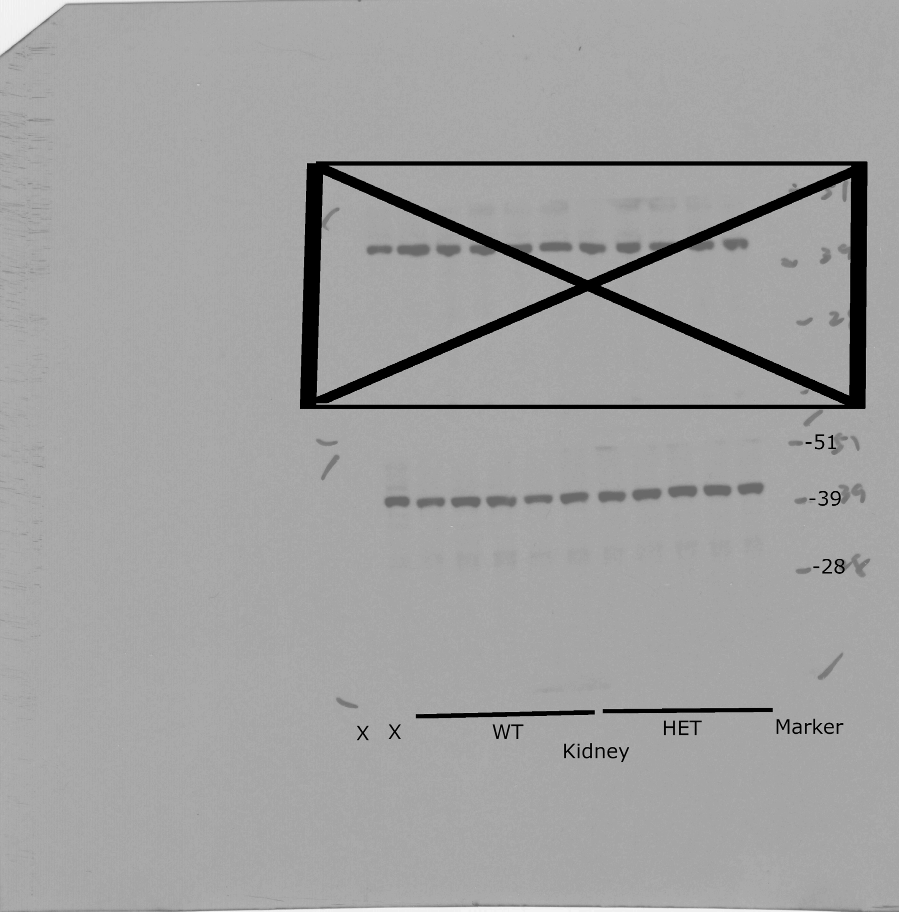

Original blot for Fig 5D actin. Kidney tissues from wild-type (WT) and hterozygous (HET)NPAT(+/-) mice were lysed in Urea lysis buffer. Samples of the lysates were run on a Novex NuPAGE 4-12% Bis-Tris gel and proteins were transferred on a PVDF membrane. SeeBlue pre-stained protein standards (LC5925) from Invitrogen were used. Actin protein signals were detected using an ECL Western blotting analysis system and a Fuji medical X-ray film. The film was scanned on a CanoScan LiDE100 scanner and the image was saved as a JPG file.

Original blot for Fig 5D ATM. Kidney tissues from wild-type (WT) and heterozygous (HET)NPAT(+/-) mice were lysed in Urea lysis buffer. Samples of the lysates were run on a Novex NuPAGE 4-12% Bis-Tris gel and proteins were transferred on a PVDF membrane. SeeBluePlus2 pre-stained protein standards (LC5925) from Invitrogen were used. ATM protein signals were detected using an ECL Western blotting analysis system and a Fuji medical X-ray film. the film was scanned on a CanoScan LiDE100 scanner and the image was saved as a JPG file.

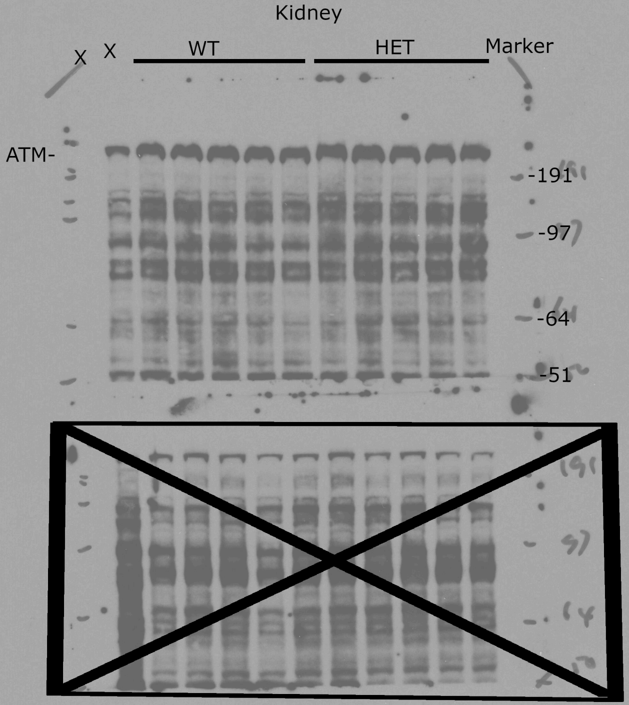

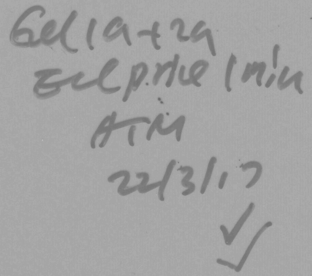

Original blot for Fig 5D NPAT. Kidney tissues from wild-type (WT) and heterozygous (HET)NPAT(+/-) mice were lysed in Urea lysis buffer. Samples of the lysates were run on a Novex NuPAGE 4-12% Bis-Tris gel and proteins were transferred on a PVDF membrane. SeeBluePlus2 pre-stained protein standards (LC5925) from Invitrogen were used. NPAT protein signals were detected using an ECL Western blotting analysis system and a Fuji medical X-ray film. The film was scanned on a CanoScan LiDE100 scanner and the image was saved as a JPG file.

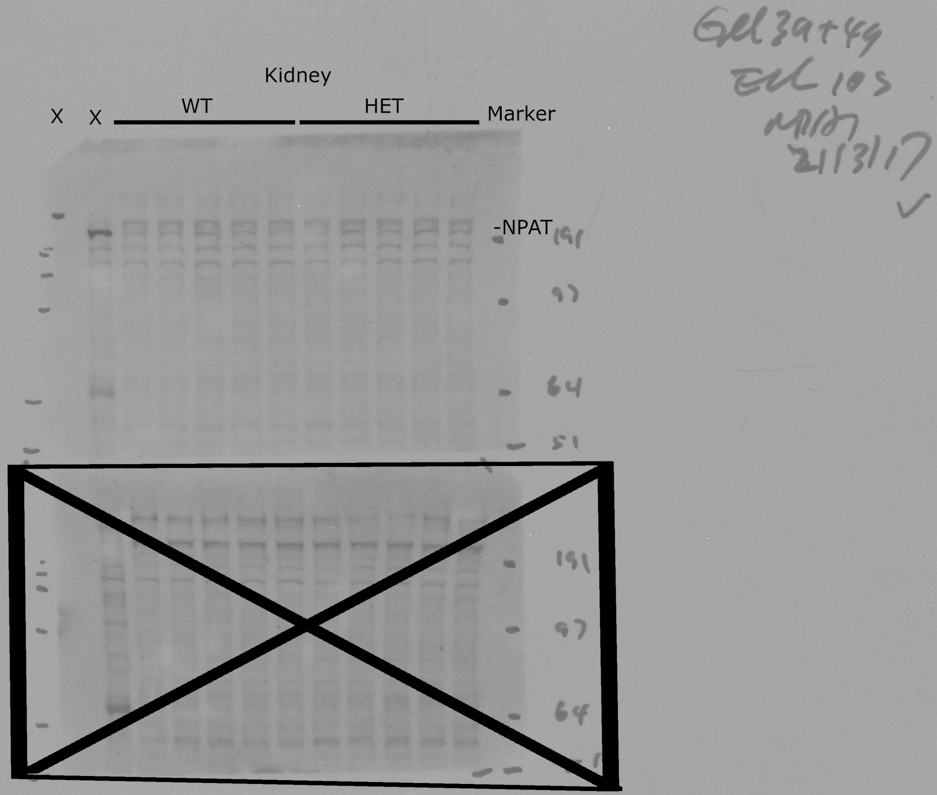

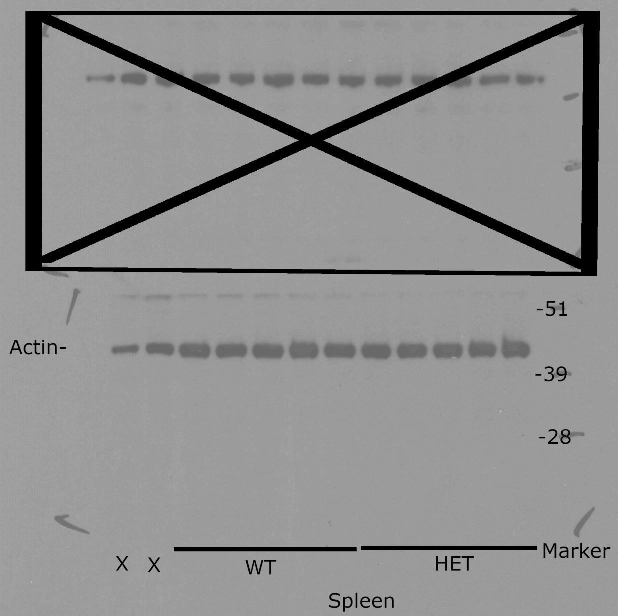

Original blot for Fig 5E actin. Spleen tissues from wild-type (WT) and heterozygous (HET) NPAT(+/-) mice were lysed in Urea lysis buffer. Samples of the lysates were run on a Novex NuPAGE 4-12% Bis-Tris gel and proteins were transferred on a PVDF membrane. SeeBluePlus2 pre-stained protein standards (LC5925) from Invitrogen were used. Actin protein signals were detected using an ECL Western blotting analysis system and a Fuji medical X-ray film. The film was scanned on a CanoScan LiDE100 scanner and the image was saved as a JPG file.

15/4/17

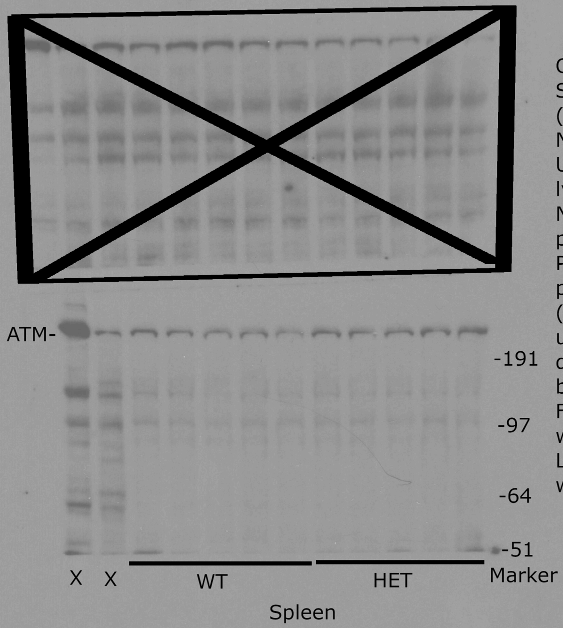

Original blot for Fig 5E ATM. Spleen tissues from wild-type (WT) and heterozygous (HET) NPAT(+/-) mice were lysed in Urea lysis buffer. Samples of the lysates were run on a Novex NuPAGE 4-12% Bis-Tris gel and proteins were transferred on a PVDF membrane. SeeBluePlus2 pre-stained protein standards (LC5925) from Invitrogen were used. ATM protein signals were detected using an ECL Western blotting analysis system and a Fuji medical X-ray film. The film was scanned on a CanoScan LiDE100 scanner and the image was saved as a JPG file.

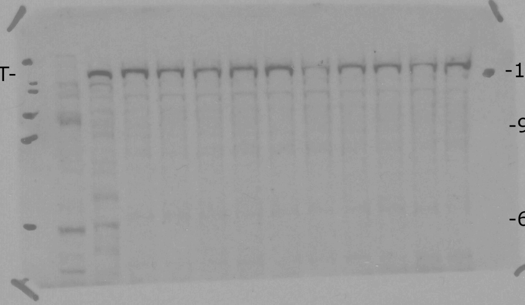

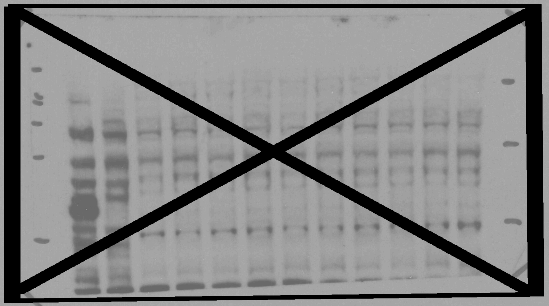

Original blot for Fig 5E NPAT. Spleen tissues from wild-type (WT) and heterozygous (HET)NPAT(+/-) mice were lysed in Urea lysis buffer. Samples of the lysates were run on a Novex NuPAGE 4-12% Bis-Tris gel and proteins were transferred on a PVDF membrane. SeeBluePlus2 pre-stained protein standards (LC5925) from Invitrogen were used. NPAT protein signals were detected using an ECL Western blotting analysis system and a Fuji medical X-ray film. The film was scaneed on a CanoScan LiDE100 scanner and the image was saved as a JPG file.
