# Supplementary material for: The genetic association of the transcription factor NPAT with glycemic response to metformin involves regulation of fuel selection
Source: PLoS One. 2021 Jul 1;16(7):e0253533. doi: 10.1371/journal.pone.0253533 (PMC8248654; doi:10.1371/journal.pone.0253533)
Supplement: S1 Table — (DOCX) [file pone.0253533.s002.docx]

**Supplemental Table S1: Source of antibodies**

| **Antibody** | **Source** | **Identifier** | **Additional information** |
| --- | --- | --- | --- |
| NPAT (C-19) antibody | Santa Cruz | Sc-32359  RRID^a)^: AB_2152225 | IF^b)^: 1: 250  IH^c)^, WB^d)^: 1: 1,000  Discontinued: 2016 |
| NPAT antibody | Bethyl Laboratories, Inc | A302-772A-M  RRID:AB_10630262 | IF: 1: 250  IH, WB: 1: 1,000 |
| Mouse Anti-NPAT monoclonal antibody, unconjugated, clone 27 | BD Bioscience | 611344  RRID:AB_398866) | IF: 1: 250  IH, WB: 1: 1,000 |
| Rabbit anti-ATM antibody | Abcam | Ab199726  RRID:AB_2722615 | IF: 1: 250  IH, WB: 1: 1,000 |
| Sheep anti-mouse IgG H&L (HRP) | Abcam | Ab6808  RRID:AB_955441 | WB: 1: 5000 |
| Donkey anti-goat IgG H&L (Alexa Fluor 647) | Abcam | Ab150131  RRID:AB_2687955 | IF:1: 1,000 |
| Donkey anti-mouse IgG H&L (Alexa Fluor488) | Abcam | Ab150105  RRID:AB_2732856 | IF:1: 1,000 |
| Donkey anti-rabbit IgG (Alexa Fluor 488) | Abcam | Ab150073  RRID:AB_2636877 | IF:1: 1,000 |
| Phospho-AMPKα (Thr172) (D4D6D) rabbit monoclonal antibody | Cell Signaling Technology | 50081  RRID:AB_2799368 | WB: 1: 1,000 |
| AMPK alpha (D5A2) rabbit monoclonal antibody | Cell Signaling Technology | 5831  RRID:AB_10622186 | WB: 1: 1,000 |
| Rabbit Anti-S6 Ribosomal Protein, phospho (Ser240 / Ser244) Monoclonal antibody | Cell Signaling Technology | 4838  RRID:AB_659977 | WB: 1: 1,000 |
| Rabbit anti-S6 ribosomal protein monoclonal antibody, unconjugated, clone 5G10 | Cell Signaling Technology | 2217  RRID:AB_331355 | WB: 1: 1,000 |
| Monoclonal anti-actin antibody produced in mouse | Sigma | A3853  RRID:AB_262137 | WB: 1: 3, 000 |
| Donkey anti-rabbit IgG, whole Ab ECL antibody, HRP Conjugated | GE Healthcare | NA934  RRID:AB_772206 | 1:20,000 |

1. RRID: Research Resource Identifier
2. IF: Immunofluorescence
3. IH: Immunohistochemistry
4. WB: Western blotting
