# Supplementary material for: The genetic association of the transcription factor NPAT with glycemic response to metformin involves regulation of fuel selection
Source: PLoS One. 2021 Jul 1;16(7):e0253533. doi: 10.1371/journal.pone.0253533 (PMC8248654; doi:10.1371/journal.pone.0253533)
Supplement: S2 Table — (DOCX) [file pone.0253533.s003.docx]

**Supplemental Table S2: Source of plasmids, vectors and transduction particles**

| **Plasmids, vectors and transduction particles** | **Source** | **Identifier** | **Additional information** |
| --- | --- | --- | --- |
| NPAT (NM_002519) human tagged ORF clone | Origene | RC220678 | Tag: Myc-DDK  Vector: pCMV6-Entry |
| [pcDNA3.1(+)Flag-His-ATM wt](https://www.addgene.org/31985/) | Addgene | 31985  RRID:Addgene_31985 | Insert:  ATM wild-type ([ATM](https://www.addgene.org/browse/gene/472/) Human)  Tags: Flag and HIS |
| [pcDNA3.1(+) Flag-His-ATM kd](https://www.addgene.org/31986/) | Addgene | 31986  RRID:Addgene_31986 | ATM kd ([ATM](https://www.addgene.org/browse/gene/472/) Human)  Tags: Flag and HIS |
| pcDNA5/RT/TO | Invitrogen | V6520-20 |  |
| pOG44 | Invitrogen | V6005-20 |  |
| pGEM-T Easy vector | Promega | A1360 |  |
| NPAT MISSION shRNA lentiviral transduction particles | Sigma | TRCN0000141527 TRCN0000276140 | Sequences of inserts in shRNA constructs targeting the NPAT gene  TRCN0000141527:  Sense:  5’-CAGCCTGCTTACTGTCCTTAT-3’  Anti-sense:  5’-ATAAGGACAGTAAGCAGGCTG-3’  TRCN0000276140:  Sense:  5’-CAGCCTGCTTACTGTCCTTAT-3’  Anti-sense:  5’-ATAAGGACAGTAAGCAGGCTG-3’ |
| MISSION pLKO.1-puro-CMV-TurboGFP positive control transduction particles | Sigma | SHC003V | A positive control to monitor transduction efficiency |
