# Supplementary material for: The genetic association of the transcription factor NPAT with glycemic response to metformin involves regulation of fuel selection
Source: PLoS One. 2021 Jul 1;16(7):e0253533. doi: 10.1371/journal.pone.0253533 (PMC8248654; doi:10.1371/journal.pone.0253533)
Supplement: S3 Table — (DOCX) [file pone.0253533.s004.docx]

**Supplemental Table S3: Source of oligos.**

| **Oligos** | **Source** | **Identifier** | **Additional information** |
| --- | --- | --- | --- |
| Primers for sequencing NPAT (NM_002519) human tagged ORF clone (RC220678) | Sigma | Customer | L1: 5’-ATGTTGTTACCCTCGGACGT-3’  L2: 5’-GAATTTCCAAGATCCAAACGCT-3’  L3: 5’-ACAACCATGATGTGCTTAGACA-3’  L4: 5’-TTCATGGTTCCAAGTCATCAGA-3’  L5: 5’-TGCATCAAATATCGTCTCTCTCA-3’  L6: 5’-GCACCTATGACTGCTCAACC-3’  L7: 5’-ACTACTGCTCCTGTGGCAAA-3’  L8: 5’-CCGCCAGTTCTTTGATTACCA-3’  R1: 5’-TCTGGCAGGTAGAAATGAGGT-3’  R2: 5’-TGAGCACCAGGAATGACATT-3’  R3: 5’-TGCTAAGACTATACTGGGATTGG-3’  R4: 5’-TGTGGCTGGCATCAACTGTA-3’  R5: 5’-TGTGCATGACATCCAACTGA-3’  R6: 5’-TTTCTCACTTCGCAAACCCC-3’  R7: 5’-GGTGCTCCTTGAAGATGCTC-3’  FOR_5: 5’-GAAGACTCTGCAGTAAACAATACTCA-3’  REV_3: 5’-CACAACCAAGTGAAACAAATGAA-3’  REV_4: 5’-TCTCACAGTTAGCATTTCCACC-3’ |
| PCR primers for cloning NPAT using RC220678 as a templet | Sigma | Customer | Forward:  5’-GCCGCCGCGATCGCC ATG TTGTTACCCTCGG-3’  Reverse:  5’-GGATCCTTAAACCTTATCGTCGTCATCCTTGTAATC-3’ |
| Primer/ probes sets for Human NPAT | ThermoFisher Scientific | Hs00159638-m1 | TaqMan gene expression assay |
| Primer/ probes sets for mouse NPAT | ThermoFisher Scientific | Mm01177407-m1 | TaqMan gene expression assay |
| Primer/ probes sets for Human ATM | ThermoFisher Scientific | Hs01112309-m1 | TaqMan gene expression assay |
| Primer/ probes sets for mouse ATM | ThermoFisher Scientific | Mm01177457-m1 | TaqMan gene expression assay |
| Primer/ probes sets for Human actin-β | ThermoFisher Scientific | Hs99999903-m1 | TaqMan gene expression assay |
| Primer/ probes sets for mouse actin-β | ThermoFisher Scientific | Mm02619580-g1 | TaqMan gene expression assay |
| siRNA for ATM: Hs_ATM_8 | Qiagen | SI00299299 | Target sequence:  5’-AACCATGAGTCTAGTACTTAA-3’ |
| siRNA for ATM: Hs_ATM_5 | Qiagen | SI00604730 | Target sequence:  5’-AAGGCTATTCAGTGTGCGAGA-3’ |
| Allstar negative control siRNA | Qiagen | SI03650318 | Target sequence is proprietary |
